# SCAG: A Stratified, Clustered, and Growing-Based Algorithm for Soybean Branch Angle Extraction and Ideal Plant Architecture Evaluation

**DOI:** 10.34133/plantphenomics.0190

**Published:** 2024-07-23

**Authors:** Songyin Zhang, Yinmeng Song, Ran Ou, Yiqiang Liu, Shaochen Li, Xinlan Lu, Shan Xu, Yanjun Su, Dong Jiang, Yanfeng Ding, Haifeng Xia, Qinghua Guo, Jin Wu, Jiaoping Zhang, Jiao Wang, Shichao Jin

**Affiliations:** ^1^ Plant Phenomics Research Centre, Academy for Advanced Interdisciplinary Studies, Collaborative Innovation Centre for Modern Crop Production cosponsored by Province and Ministry, State Key Laboratory of Crop Genetics and Germplasm Enhancement, Nanjing Agricultural University, Nanjing 210095, China.; ^2^ National Center for Soybean Improvement, Key Laboratory for Biology and Genetic Improvement of Soybean (General, Ministry of Agriculture), College of Agriculture, Nanjing Agricultural University, Nanjing 210095, China.; ^3^ Sanya Research Institute of Nanjing Agriculture University, Sanya 572024, China.; ^4^ State Key Laboratory of Vegetation and Environmental Change, Institute of Botany, Chinese Academy of Sciences, Beijing 100093, China.; ^5^ School of Automation, Southeast University, Nanjing 210096, China.; ^6^ Institute of Remote Sensing and Geographic Information System, School of Earth and Space Sciences, Peking University, Beijing 100871, China.; ^7^ Division for Ecology and Biodiversity, School of Biological Sciences, The University of Hong Kong, Hong Kong, China.

## Abstract

Three-dimensional (3D) phenotyping is important for studying plant structure and function. Light detection and ranging (LiDAR) has gained prominence in 3D plant phenotyping due to its ability to collect 3D point clouds. However, organ-level branch detection remains challenging due to small targets, sparse points, and low signal-to-noise ratios. In addition, extracting biologically relevant angle traits is difficult. In this study, we developed a stratified, clustered, and growing-based algorithm (SCAG) for soybean branch detection and branch angle calculation from LiDAR data, which is heuristic, open-source, and expandable. SCAG achieved high branch detection accuracy (*F-score* = 0.77) and branch angle calculation accuracy (*r* = 0.84) when evaluated on 152 diverse soybean varieties. Meanwhile, the SCAG outperformed 2 other classic algorithms, the support vector machine (*F-score* = 0.53) and density-based methods (*F-score* = 0.55). Moreover, after applying the SCAG to 405 soybean varieties over 2 consecutive years, we quantified various 3D traits, including canopy width, height, stem length, and average angle. After data filtering, we identified novel heritable and repeatable traits for evaluating soybean density tolerance potential, such as the ratio of average angle to height and the ratio of average angle to stem length, which showed greater potential than the well-known ratio of canopy width to height trait. Our work demonstrates remarkable advances in 3D phenotyping and plant architecture screening. The algorithm can be applied to other crops, such as maize and tomato. Our dataset, scripts, and software are public, which can further benefit the plant science community by enhancing plant architecture characterization and ideal variety selection.

## Introduction

Soybean is a major source of plant oil and protein used for human consumption. The burgeoning global population demands a large increase in soybean production [1]. Increasing soybean productivity is, therefore, a long-term breeding demand. Because the area of arable cropland is limited, increasing crop productivity per unit of land area is a widely recognized breeding target. Therefore, it is urgent to screen density-tolerant soybeans, discover new genes controlling plant architecture, and design/breed elite density-tolerant soybeans.

Plant architecture is a comprehensive trait that characterizes crop production potential and is widely recognized as the main direction of high-yield breeding [2]. Plant architecture refers to the 3-dimensional (3D) organization of a plant, including stem height, branching pattern, and the shape and position of leaves and reproductive organs [3]. The success of the “Green Revolution” for rice and wheat inspired us to improve yield by designing a semidwarf plant architecture. Unlike grain crops that have panicles/heads above the canopy, soybean is a typical pod crop whose yield organ grows throughout the plant [4]. With respect to the “Green Revolution” for soybean plants, one key aspect of plant architecture is branch formation, particularly the branch angle, which directly determines canopy coverage and further influences plant functions, especially light interception, photosynthetic efficiency, and yield [5,6]. Smaller branch angles contribute to narrow architecture, enabling dense planting [4,7,8]. More importantly, small (vertical) angles at the top of the plant and large (horizontal) angles at the bottom can maximize the light interception of an individual plant and optimize the light distribution and utilization of a crop population, forming a “smart canopy” [9] for high-yield production. Therefore, the branch angle is vitally important for evaluating soybean density tolerance and yield potential.

High-throughput branch phenotyping is one of the key challenges for 3D plant architecture studies. The traditional methods for branch angle measurement mainly rely on manual operations, such as the use of inclinometers [10], which are tedious and difficult to perform. Two-dimensional image-based techniques have improved the efficiency and precision of branch angle acquisition [11]. For example, image-based leaf angle extractors were developed to improve the efficiency of leaf angle extraction in maize and sorghum [12]. However, these methods are mainly focused on crops (e.g., maize and sorghum) with simple canopy structures (e.g., symmetrical distribution of leaves) and require specifically selected shooting angles. In contrast, soybean crops have more complex 3D plant architecture, thus making it difficult to determine an optimal shooting angle.

Light detection and ranging (LiDAR) is an active remote sensing technique used to directly acquire the 3D information of an object, shedding new light for 3D plant phenomics [13,14]. LiDAR is superior to other 3D reconstruction techniques, such as stereo imagers [15] and depth cameras [16], which are easily affected by environmental conditions (e.g., illumination) [17–19], object surface properties (e.g., continuity and flatness) [20], and camera calibration [21–23]. LiDAR has been widely applied to phenotypic tasks due to its high accuracy and insensitivity to environmental light ([24–29]). However, LiDAR point cloud-based methods have been mainly developed for crops with simple structures, such as for calculating the leaf angles in maize and sorghum [30–34]. These methods can be categorized into 2 major types: geometric methods and machine learning-based methods. Geometric methods usually use the geometric distance between the samples in the point clouds [30] and the curvature calculation method [32] for skeleton extraction and trait calculation. Notably, geometric methods mostly rely on handcrafted features and expert experiences. In contrast, machine learning-based methods automatically learn features based on given abundant data samples and their corresponding labels. The labeled key points of plants allow for machines to learn the rules for expected targets and automatically recognize plant phenotypic traits [35]. However, it is difficult to interpret machine learning-based methods and apply them to other scenarios (e.g., crop types). Therefore, geometric algorithms are usually selected by the research community if effective features and rules can be designed and large-volume datasets are unavailable.

Branch detection algorithms are critical for extracting complex 3D branch angles and empowering further 3D phenomics applications. Skeleton extraction is the most frequently used method for branch angle detection based on point clouds [36]. The Laplacian contraction algorithm has been widely used in skeleton extraction by shrinking point clouds, such as in maize [34]. In addition, several heuristic methods have been developed [30,32]. The MNVG (median normalized-vector growth) method was proposed for generating the skeleton by calculating the median points of a ball region from the bottom upward [30]. Similarly, the curvature calculation method was developed for extracting the skeleton of maize by finding points with large curvatures [32]. Notably, skeleton-based methods are effective for large crops with simple canopy structures. However, these methods are not applicable to soybean plants due to various challenges: (a) Complex 3D structure: The branches of soybean plants are distributed throughout 3D space, thus making their structure more complex than that of crops with leaves almost symmetrically distributed along a main stem, such as maize and sorghum. (b) Fewer curved branching organs: Plant architecture is closely related to branching organs. Compared with the flexible leaves of maize and sorghum, the branches of soybean plants are wood-like with no obvious curvature. (c) Smaller crop size: The average height, stem width, and branch width of soybean are smaller than those of other crops, such as maize, thus leading to a smaller signal-to-noise ratio in soybean point cloud data under the same scanning specifications.

The main objectives of this study are presented below.

1. To develop a new soybean branch detection algorithm, i.e., the stratified, clustered, and growing-based algorithm (SCAG), based on LiDAR data.

2. To evaluate the performance and robustness of the proposed algorithm through model comparison and parameter sensitivity analysis.

3. To verify the applicability and transferability of the proposed algorithm to other crop types and its value in evaluating the potential of soybean density tolerance based on plant architecture (e.g., branch angles).

The proposed SCAG could facilitate high-throughput soybean branch angle extraction and accelerate branch angle-based plant architecture screening, which is of great interest to the breeding community.

## Materials and Methods

### Study area and data collection

**Fig. 1.**
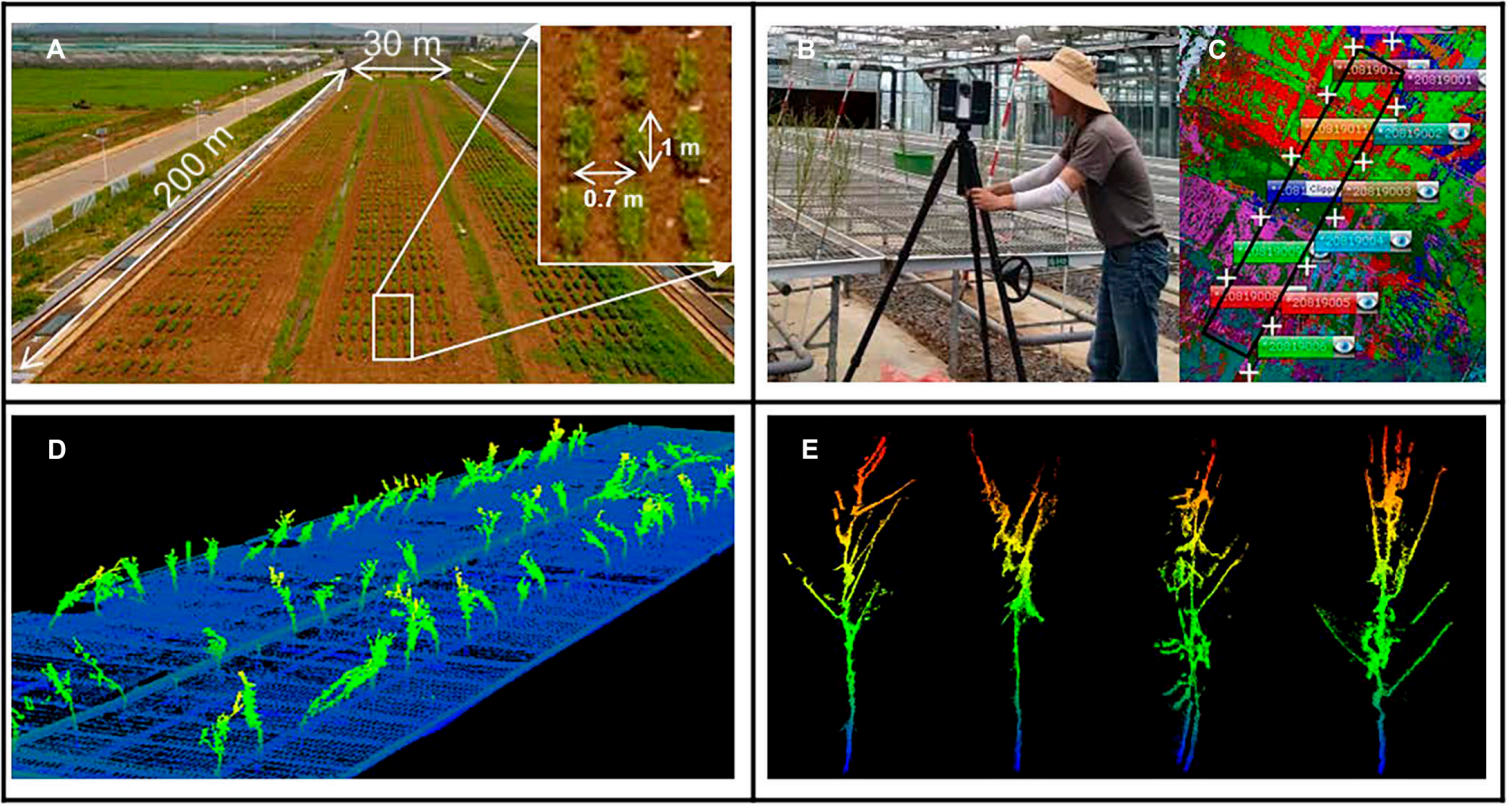
The study area and process of data collection. (A) The experimental field was approximately 200 m by 30 m. Each plot consisted of 3 rows of the same soybean variety, with a row width of 1 m and a row spacing of 0.7 m. The field contained 810 plots, which included 2 replications of 405 soybean varieties. (B) Soybean plants were fixed on an indoor platform and scanned by using a TLS. (C) Point cloud data were registered using the SCENE software, where the black box is the platform and the white “+” represent the TLS locations. (D) An overview of the collected LiDAR data on 2021 October 22. (E) The point cloud data of several representative soybean varieties.

The study area was located at the Baima Experimental Station of Nanjing Agricultural University in Nanjing, Jiangsu Province, China (119°18′71″E, 31°62′00″N). A 2-year germplasm screening experiment was repeatedly conducted in both 2021 and 2022. The length and width of the experimental field were approximately 200 and 30 m, respectively. There were 810 plots in the field that included 2 replications of 405 soybean varieties (Fig. 1). Each plot had 3 plant rows with a row spacing of 0.7 m and a row length of 1 m. The distance between adjacent plants within a row was approximately 0.07 m. Two border rows were planted around the target plots for protection. These varieties have remarkable diversity and representativeness in terms of plant architecture and were selected from all over the world by the National Center for Soybean Improvement (https://ncsi.njau.edu.cn/sysgk1/sysjj.htm).

Although we aimed to assess soybean plant architecture in real production conditions, it was difficult to acquire high-quality individual point clouds in a changeable field environment with a dense canopy. Therefore, 3 plants of each variety per plot were randomly selected from the field and transferred to an indoor platform for scanning to ensure data accuracy during the maturity stage. The leaves of plants at the maturity stage were manually defoliated before scanning in 2021, and the leaves and pods were removed before scanning in 2022. The soybean individuals of different varieties were fixed in rows with a row spacing of approximately 0.7 m and a plant spacing of approximately 1 m on a platform with a length of 23 m and a width of 3.5 m (Fig. 1B). The point clouds of these varieties are scanned by using a terrestrial LiDAR scanner (TLS, Faro Focus3D S70) at multiple stations. The scanning time of each station was approximately 4 min, and the point cloud resolution was approximately 2 mm. There were 10 to 14 stations in the platform area, which were distributed along the edge of the platform, and the distance between 2 stations was approximately 3.7 m (Fig. 1C). Finally, these multiple stations of TLS data were registered into a whole point cloud using SCENE software.

### Data preprocessing and dataset construction

In both 2021 and 2022, the registered LiDAR data were manually segmented into 3,218 individual point clouds of 405 varieties (each variety had 1 to 2 individual samples) and denoised using the statistical outlier removal algorithm in *CloudCompare* software. A total of 152 individual samples were randomly selected to construct a well-labeled dataset (Soybean3D) for algorithm development (see details in Supplemental Materials S1). The Soybean3D dataset is diverse (Fig. S1) and can be further divided into 3 different complex groups based on the number of branch angles and average branch angle (Table S1).

### Algorithm design of the SCAG

**Fig. 2.**
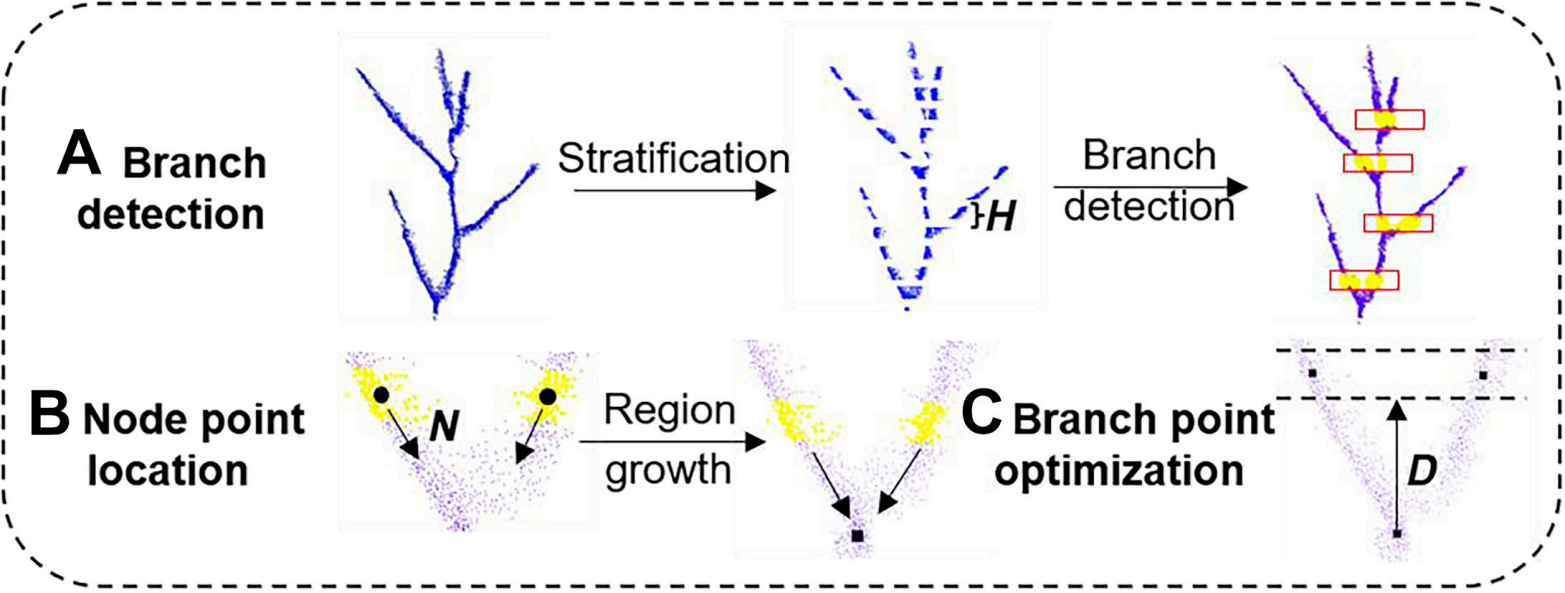
An overview of the proposed method. (A) Branch detection: The soybean point cloud was sliced into multiple layers of the same height. The branch locations were detected based on the cluster relationships of adjacent layers. (B) Node point location: The node point was located by using the downward region growth method based on the branch points detected in the previous step. (C) Branch point optimization: Two branching points were relocated by slicing a new upward layer that was closer/similar to manual operations from the node point. The optimized branch points and node points were used to calculate branch angles.

This work presents an SCAG framework for branch angle detection and calculation. The SCAG includes 3 steps, i.e., branch detection, node location, and branch point optimization (Fig. 2). The branch detection step was designed to coarsely locate the branches from a series of point clouds of a slice height (*H*) from the bottom to the top. The node point location step aims to find node points (connection points between stems and branches) by using the downward region growth method based on the above-detected branch points. Branch point optimization is a postprocessing step for optimizing the above-detected branch locations that may be too close to the node points. The angle calculation uncertainty can be decreased based on the finely optimized branch points and node points.

Step 1: Branch detection

Branch detection aims to coarsely find the locations of the branches for branch angle calculation, which requires finding 2 branch points and 1 node point. The parameter slice height (*H*) was used to slice a point cloud into different layers from the bottom to the top because the branches were located at different heights. The point cloud of each layer was clustered by using density-based spatial clustering of applications with noise (DBSCAN) [37]. The branch locations were detected by analyzing the number of clusters and the relationship between any 2 adjacent layers. The number of clusters in each layer and its next bottom layer mainly included 3 cases.

Case 1: The number of clusters in the upper slice layer (*CNU*) was greater than the number of clusters in the lower slice layer (*CNL*), i.e., *CNU* > *CNL*.

**Fig. 3.**
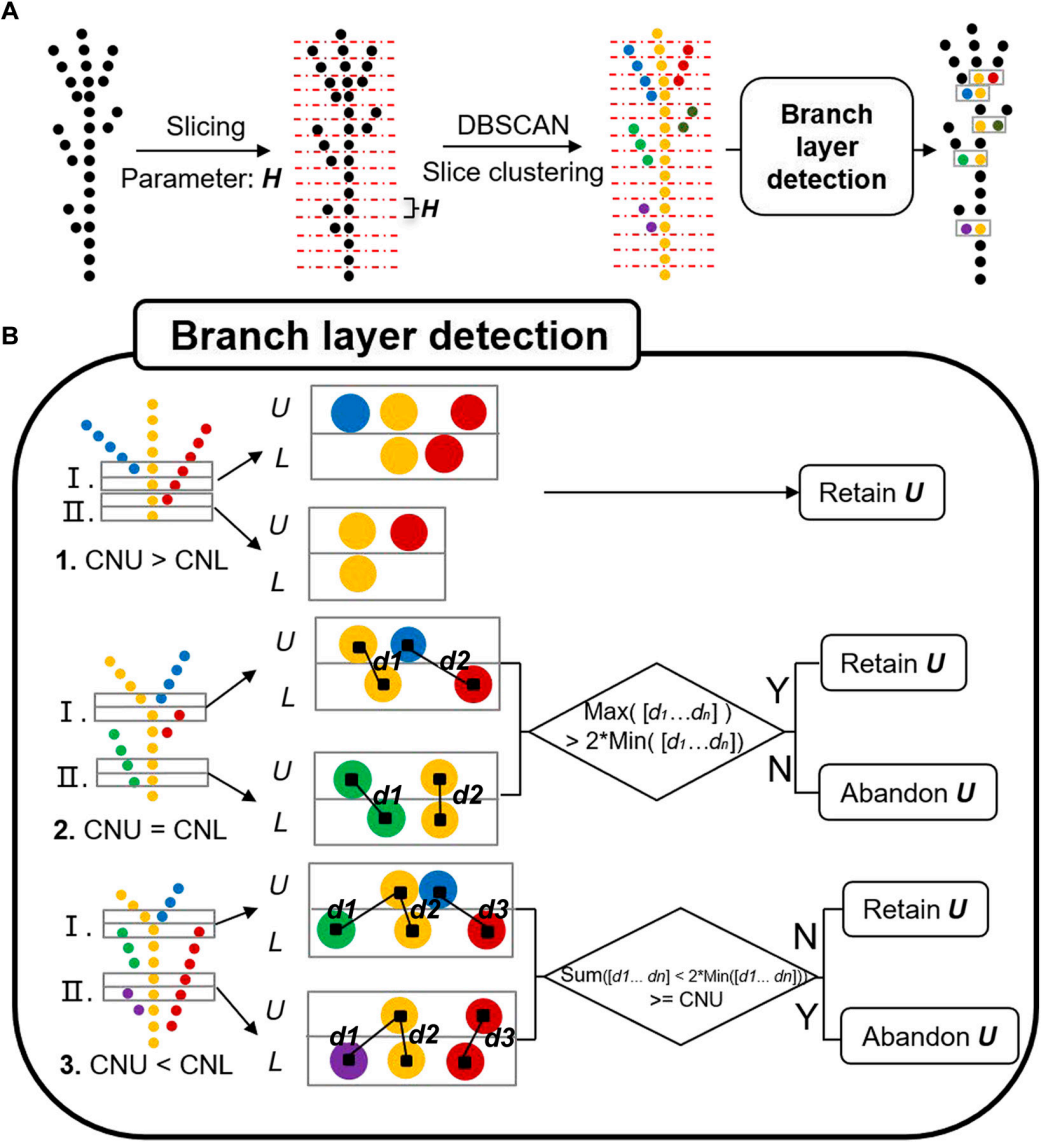
Flowchart of slice clustering and branch detection. (A) Branch detection workflow. The parameter slice height (*H*) represents the height of the slice. (B) The logic for detecting the branch layers is based on the number of clusters in 2 adjacent layers. *U* and *L* represent the upper and lower layers, respectively. *CNU* and *CNL* represent the number of clusters in the upper and lower layers, respectively. *dn* (e.g., *d1, d2*, and *d3*) represents the nearest distance from a cluster in the lower layer to a cluster in the upper layer.

This was the simplest case, i.e., the number of clusters increased from the bottom to the top. This case meant that a new branch was formed, as presented in Fig. 3B1. Therefore, the clusters in the *CNU* were preserved for locating branches.

Case 2: The number of clusters in the upper slice equaled the number of clusters in the lower slice, i.e., *CNU* = *CNL*.

This case included 2 possible results: (I) the upper layer contained a newly split branch (Fig. 3B, 2, I), which should be retained; and (II) the upper layer did not contain a newly split branch but rather a continuation of the previous branch (Fig. 3B, 2, II), which should be ignored.

To determine if a layer contained a new branch, a distance-based rule was proposed by considering the distances (i.e., *D_CNL_* = [d_1_, …, d_CNL_]) between each cluster in the lower layer and its nearest cluster in the upper layer. Each cluster was represented by a median point calculated from points belonging to the cluster. We used the median value instead of the mean value of the point cloud as the cluster center because our previous findings indicated that the median value is insensitive to noisy points [30]. If one of the clusters in the lower layer did not have a neighboring cluster within the given distance threshold in the upper layer, then the upper layer contained new branches (Fig. 3B, 2, I); otherwise, if each cluster in the lower layer had neighboring clusters within the given distance threshold in the upper layer, the upper layer did not contain a new branch but a continuation of a lower branch (Fig. 3B, 2, II). This biological observation can be formulated into a distance-based rule, i.e., if the maximum value of the nearest distances (i.e., *max (D_CNL_)*) is greater than twice the minimum value (i.e., *min (D_CNL_)*), the upper layer should be retained for locating the new branch. Otherwise, the upper layer was considered a continuation of the bottom branch and was discarded. The “twice” threshold was selected according to repeated trials and errors.

Case 3: The number of clusters in the upper slice is smaller than the number of clusters in the lower slice, i.e., *CNU* < *CNL*.

This case also includes 2 possible results: (I) the upper layer contained a new branch (Fig. 3B, 3, I), which should be retained; (II) the upper layer did not contain a newly split branch but rather a continuation of several previous branches (Fig. 3B, 3, II), which should be ignored.

To determine if a layer contained a new branch, the proposed distance-based rule was used. First, the distances between each cluster in the lower layer and its nearest neighbor cluster in the upper layer were calculated, i.e., *D_CNL_*. Afterward, the number of elements in *D_CNL_* that satisfied the rule of being smaller than 2 times *Min(D_CNL_)* was counted and named *Nmin*. When *Nmin* was less than *CNU*, some clusters in the upper layer were unable to find their source branches in the lower layer. Here, the upper layer contained new branches (Fig. 3B, 3, I). In contrast, if *Nmin* was equal to or greater than *CNU*, then all the clusters in the upper layer could find their source branch in the lower layer, which meant that the upper layer had no new branches (Fig. 3B, 3, II).

Step 2: Node point location

**Fig. 4.**
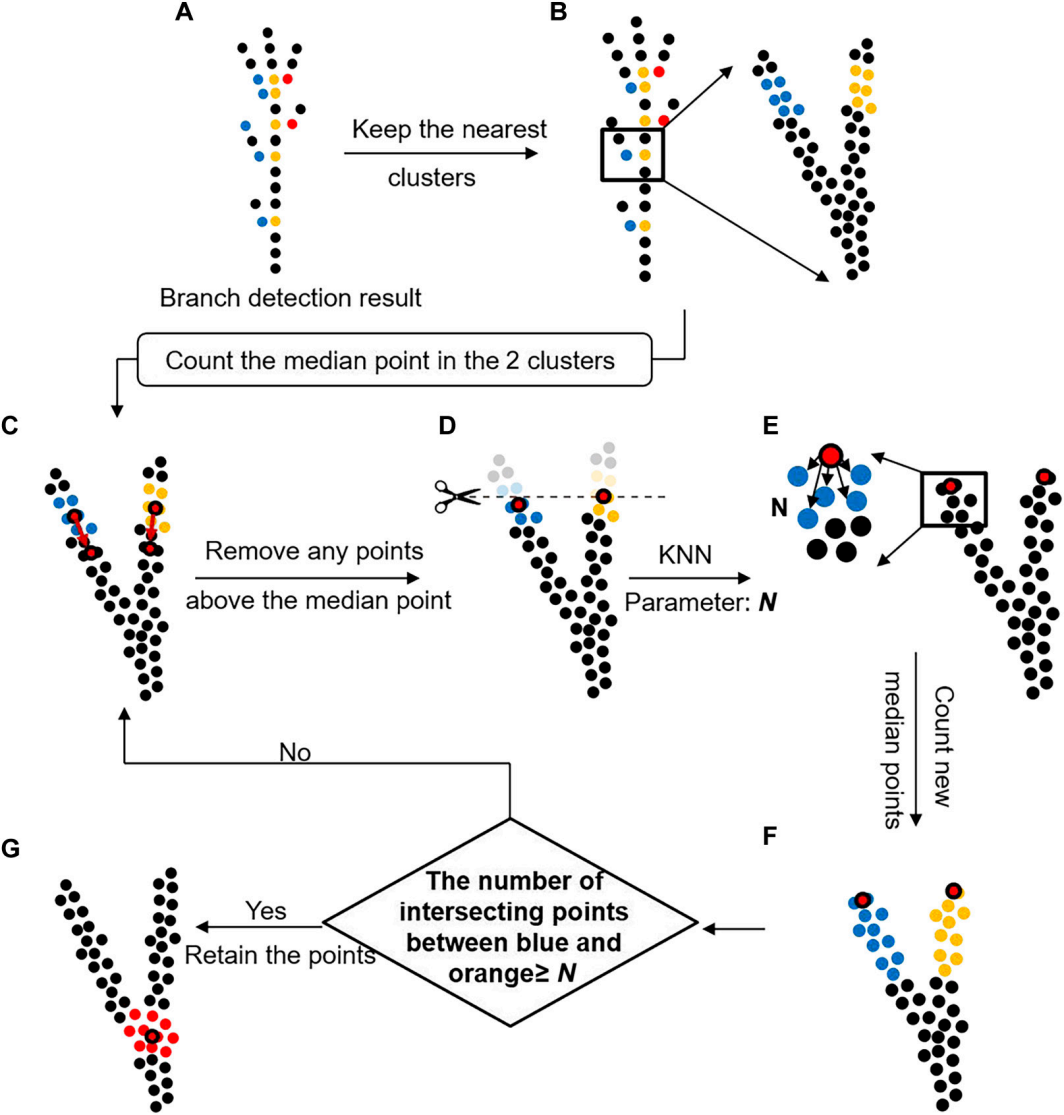
Flowchart of the node location and downward regional growth. (A) The layers include new branches that are marked with colorful points after branch detection. (B) The 2 closest clusters were kept in each layer. (C) The median points of the 2 clusters were calculated. (D) Points greater than the median point were removed. (E) The K-nearest neighbor (KNN) method was used to search for *N*-nearest neighbor points with 2 median points as the seed points. (F) The 2 searched new clusters are presented in yellow and blue. If the number of points (overlapping points) obtained during the search on both sides was greater than *N,* the 2 clusters were used to extract new median points, which were then used for repeating steps (C) to (F). Otherwise, (G) the downward search ended, and the median of the overlapping points was extracted as the node point.

The node point location was designed to find the intersection point of the main stem and branches (Fig. 4). It was implemented by considering regional growth based on the detected branch clusters. As the clusters forming a branch angle were often the closest clusters, this study considered only 2 clusters belonging to a new branch by finding the 2 closest clusters in each layer, although there was a possibility of more than 2 clusters existing in the same layer. The median values of 2 detected clusters were calculated and used as the seed points in the 3 steps of the downward regional growth method. First, the points with z values larger than the 2 seed points were removed from the point cloud of the plant. Second, the 2 seed points (on the main stem and the branch) were used for searching for the *N*-nearest neighbors, i.e., growth point number, with the *KD-Tree* [38] searching method. The resulting *N* points on each side were extracted as a new cluster, which was then used to recalculate the new seed points. Third, the new seed points were used to repeat the aforementioned downward growth process, i.e., the first 2 steps. The growth ended when the number of points (overlapping points) obtained on both sides during the search was greater than *N*. Finally, the overlapping points were extracted, and their median was used as the node point.

Step 3: Branch point optimization

**Fig. 5.**
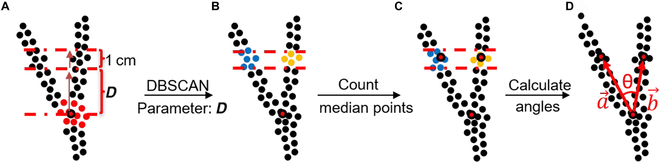
Branch point optimization and branch angle calculation. The parameter slice depth (*D*) denotes the distance used for upward optimization based on the node point. (A) The points from a 1-cm-deep layer were selected above the branch point with a height of *D*. (B) The selected points were clustered into 2 groups using DBSCAN. (C) The median points of 2 clusters were calculated, which represented the 2 branch points after optimization. (D) The branch angle was calculated based on 2 spatial vectors formed by the optimized branch points and the node point.

Branch point optimization was designed to improve the angle calculation accuracy by finely adjusting the locations of branch points. As the branch points obtained by slicing and branch detecting were too close to the node point, the angle calculation had large uncertainties based on the measuring technique. The branch points can be optimized based on the distance between them and the newly founded node point. First, we selected a layer above the node point with a thickness of 1 cm and a slice depth (*D*) for simulating the manual measurement. Second, DBSCAN was used for dividing the points in the sliced layer into 2 groups and calculating the medians of each group as the optimized branch points. Third, 2 spatial vectors, i.e., $\vec{a}$ and $\vec{b}$, were formed by connecting the node point and the optimized branch points, which were used for calculating the branch angle (Fig. 5D).

### Accuracy assessment of the proposed SCAG

The accuracy assessment of the proposed SCAG mainly consisted of 2 parts, namely, branch detection accuracy assessment and angle calculation accuracy assessment. The branch detection accuracy was evaluated by analyzing the Euclidean distance between the SCAG-predicted node points and the manually labeled node points. If there was one or more labeled node points surrounding the predicted node point with a distance of less than 1 cm (approximately the average diameter of a soybean node area), the prediction was correct and was called a true-positive (*TP*) case; otherwise, the prediction was treated as a false-positive (*FP*) case. If there was no predicted node point in the 1-cm neighborhood of a labeled node point, the node point was missed and called a false negative (*FN*). Finally, the *recall* (*R*), *precision* (*P*), and *F-score* (*F*) [39] were calculated. The values of all 3 metrics range from 0 to 1. A larger value indicates better accuracy. These metrics can be mathematically expressed as follows:

$$R=\frac{\mathrm{TP}}{\mathrm{TP}+\mathrm{FN}}$$ (1)

$$P=\frac{\mathrm{TP}}{\mathrm{TP}+\mathrm{FP}}$$ (2)

$$F=2\times \frac{R\times P}{R+P}$$ (3)

The angle calculation accuracy was evaluated by using the coefficient of correlation (*r*) and root mean squared error between the SCAG predictions and manual measurements.

### Parameter sensitivity analysis of SCAG

The proposed SCAG has 3 important parameters, namely, the slice height (*H*), growth point number (*N*), and slice depth (*D*). Theoretically, the branch detection accuracy may be influenced by parameters *H* and *N*, whereas parameter *D* may influence only the accuracy of the branch angle calculation.

The sensitivity analysis of parameters *H* and *N* was evaluated with the *F-score* under different parameter combinations using a grid-searching method in Python. The parameter *H* was set as 0.5, 1, 1.5, 2, 2.5, and 3 cm relative to the height of the adjacent branching node. The parameter *N* was chosen from 20, 30, 40, 60, 80, 100, 120, and 140 by considering the point density around the branching node. Similarly, the sensitivity analysis of parameter *D* was evaluated with the correlation coefficient *r* under different selections using an equally spaced searching method in Python. Parameter *D* was chosen from 1.5, 2, 2.5, 3, 3.5, and 4 cm based on manual measurements.

### Cross-comparison with other methods

The proposed SCAG was compared with 2 other methods, including the heuristic method (i.e., a threshold method based on point density) and the machine learning-based method (i.e., support vector machine [SVM] [40]). These 3 methods mainly differ in terms of the techniques used for detecting the branches, but they use the same approach for calculating the angles. The density-based (DB) method is a heuristic method that is based on the knowledge that the local point density of a branch node point is greater than that of other locations. In contrast, the SVM method performs point classification (i.e., node point or not) in a supervised manner based on manually labeled targets and handcrafted features. The reasons for selecting SVM for comparison were based on its wide usage and low dataset requirements [41]. The implementation details of the 2 methods are provided in Supplemental Materials S2.

## Results

### The SCAG performed well in branch detection and branch angle calculation

**Fig. 6.**
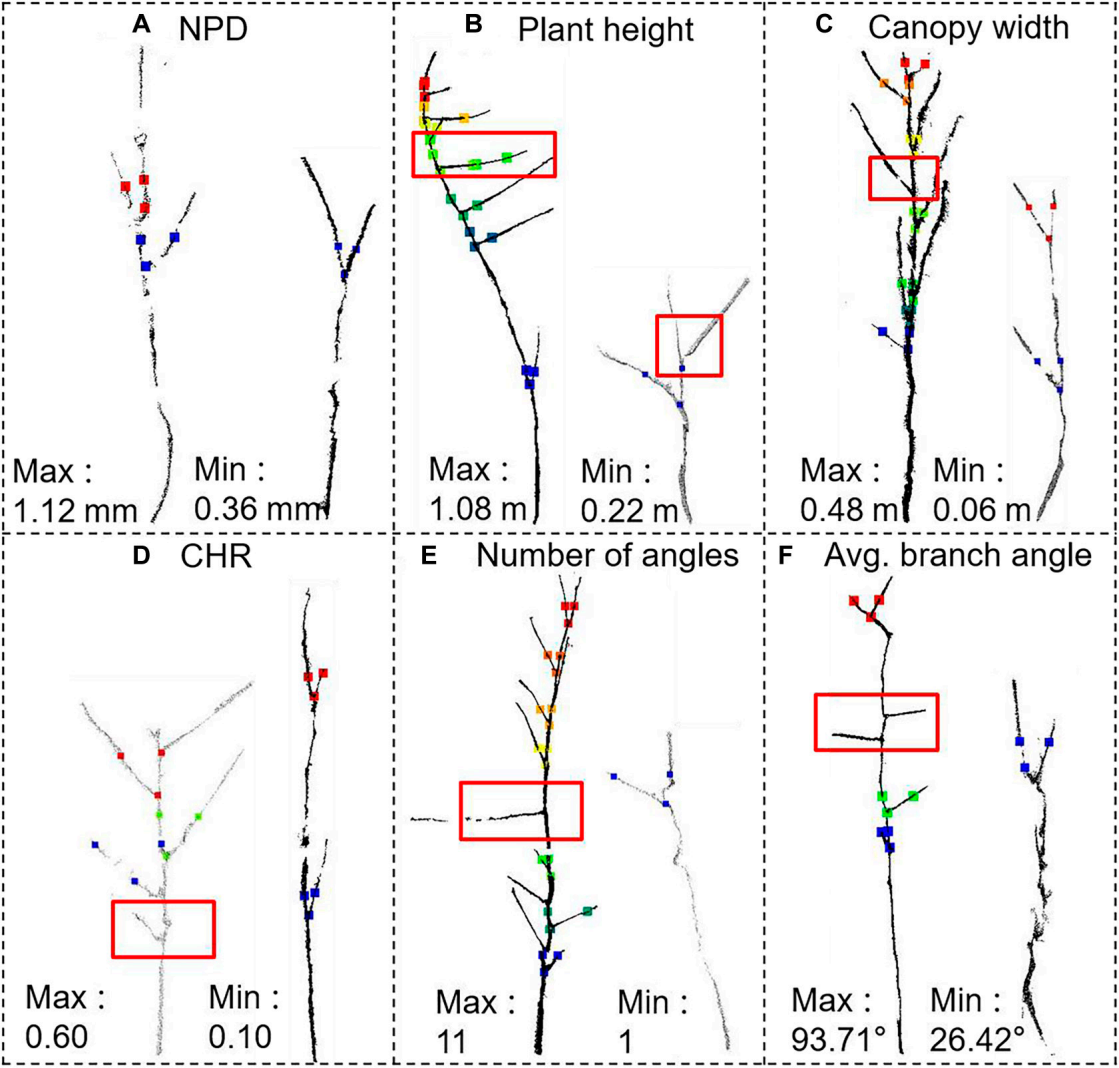
Branch detection and node location results for 12 representative soybean varieties with high phenotypic diversity. The 3 points in different colors on each soybean crop represent 1 node point and 2 branch points of the branch detected by the SCAG. The left and right subplots represent the point clouds of the plants with the largest and smallest (A) NPD, (B) plant height, (C) canopy width, (D) CHR, and (E) number of branch angles, respectively. (F) Average branch angles. The red boxes indicate branches not detected by the algorithm.

The proposed SCAG was evaluated qualitatively and quantitatively by using the Soybean3D dataset. Twelve representative samples were selected to visually demonstrate the branch detection performance due to limited space (Fig. 6). The samples included maximum and minimum values of the plant height, canopy width, ratio of canopy width to height (CHR), average angle, number of branch angles, and nearest point distance (NPD). In each subplot, each detected branch consisted of 3 points of the same color, which represented 2 branch points and 1 node point. NPD had a minor effect on SCAG performance because branches were accurately detected in large and small NPD cases (Fig. 6A). Similarly, the proposed SCAG accurately detected the branch angles of samples with different plant height, canopy width, CHR, branch angle number, and average angle values. Some underdetections existed when the points were missing (Fig. 6C) or the branch angle was too large (Fig. 6E and F). Overall, the proposed SCAG performed effectively for different soybean varieties.

The quantitative results showed that the mean *R*, *P*, and *F-score* of branch detection were 0.81, 0.73, and 0.77, respectively (Table 1). Moreover, the *F-scores* of the simple, medium, and complex groups were 0.80, 0.77, and 0.66, respectively. Furthermore, the quantitative results of the branch angle calculations showed that the mean *r* of the proposed SCAG was 0.84. The *r* values for the simple, medium, and complex samples were 0.88, 0.85, and 0.83, respectively (Supplemental Materials S2), which showed a decreasing trend. In addition, the proposed SCAG-predicted angles showed a slight overestimation for small angles and an underestimation for large angles in the different complex groups.

**Table 1.** The accuracy of branch detection and angle calculation for the proposed SCAG based on the Soybean3D dataset

Group	Sample number	Recall	Precision	F-score	*r*
Simple	50	0.75	0.86	0.80	0.88
Medium	51	0.77	0.78	0.77	0.85
Complex	51	0.71	0.62	0.66	0.83
All	152	0.81	0.73	0.77	0.84

### The SCAG was superior to the other methods and was robust

For branch detection, the proposed SCAG detected most of the angles (*R* = 0.81), which was better than the SVM (*R* = 0.52) and DB (*R* = 0.55) methods (Table 2). Moreover, SCAG outperformed the other 2 methods in terms of the *precision* (0.73) and *F-score* (0.77). This showed that the proposed SCAG detects more branches with higher *precision*. In the angle calculation, all 3 methods achieved high performance with *r* values greater than 0.80, and the differences among the 3 methods were relatively small.

**Table 2.** The best results of the 3 calculation methods for branch angle detection after parametric sensitivity analysis

Method	Recall	Precision	F-score	*r*
SVM	0.52	0.55	0.53	0.83
DB	0.55	0.56	0.55	0.88
SCAG	0.81	0.73	0.77	0.84

**Fig. 7.**
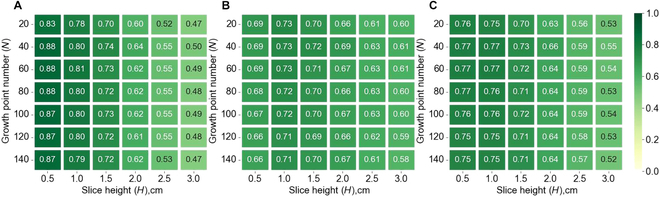
Parameter sensitivity analysis of branch detection. (A to C) *Recall*, *precision*, and *F-score* of branch detection under various parameter combinations of slice height (*H*) and growth point number (*N*).

Parameter sensitivity analyses were separately conducted for branch detection and angle calculation. The branch detection was insensitive to parameter *N* but influenced by parameter *H* following a clear pattern (Fig. 7). On the one hand, the optimal value of *H*, according to the best *F-score* (0.77; Fig. 7C), was between 0.5 and 1.0 cm, which was around 2 times of the branch diameter. On the other hand, the *R* value decreased with *H* (Fig. 7A), and the *P* value first increased and then decreased (Fig. 7B).

**Fig. 8.**
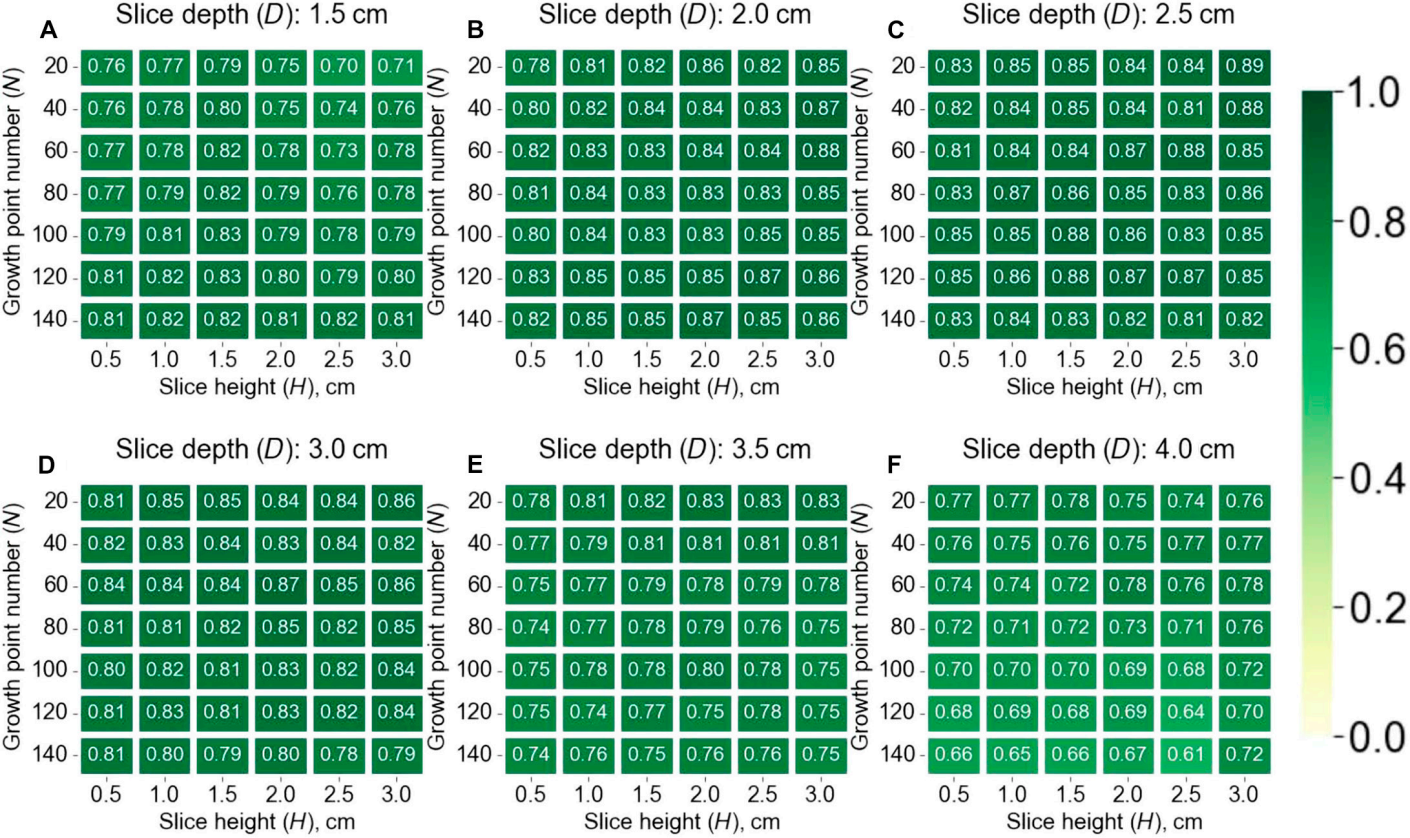
Parameter sensitivity analysis of the branch angle calculation according to the angle calculation accuracy (*r*). (A to F) The title of each subplot denotes the value of parameter *D*. The horizontal and vertical coordinates of each subplot represent the parameters *H* and *N*, respectively.

In the angle calculation, *D* was the only parameter that affected the result. However, the angle calculation accuracy may be indirectly influenced by the branch detection process, which is influenced mainly by parameter *H* but is insensitive to parameter *N* according to Fig. 7. Therefore, we analyzed the influences of parameters *D* and *H* on the angle calculation. The results showed that *H* has only a subtle influence. The *r* was relatively higher when *D* was approximately 2.0 to 3.0 cm (Fig. 8), which was closer to the threshold height between branch points and node points when measured manually.

## Discussion

### Automatic, accurate, and robust branch detection and angle calculation

This study proposed an accurate method (SCAG) for branch detection inspired by the growth laws of plants. The proposed SCAG is accurate in most cases (Fig. 6 and Table 1). Some underdetection cases were mainly caused by missing data due to overlapping (Fig. 6C), too short branches (Fig. 6D), or too large branch angles that do not obey the principle based on the upward growing angle (Fig. 6E and F). On the other hand, overdetection cases are rare unless excessive noise points exist.

In addition to high accuracy, the proposed SCAG is not very sensitive to parameter *N* within the range of 20 to 140 or parameter *D* within the range of 1.5 to 4.0 cm, and it is affected by parameter *H* (i.e., slice height in branch detection). (Fig. 7). If *H* is too large, a slice layer may include multiple node points, resulting in a lower *recall* (Fig. 7A). If *H* is too small, a slice may have too few points, which may be considered noise during the clustering process, thus reducing *precision* (Fig. 7B). Theoretically, the proposed SCAG should be robust because it detects branches based on the logic of branch growth based on clustered median points instead of considering every point. This robustness of the median points has been discussed in previous studies [30,42]. In practice, the parameter *H* should be set to approximately 2 times the branch diameter according to the sensitivity analysis (Fig. 7). Parameter *D* is suggested to be consistent with manual measurements (Fig. 8). The parameter *N* is insensitive and can be set by simple pretesting (Fig. 7).

The proposed SCAG outperforms the SVM and DB methods (Table 2) because it is difficult to define a feature set for SVM and a robust density threshold for the DB method, given a sparse, unordered, and unstructured point cloud. In the DB method, the reason for the low angle detection rate is that the density of the point cloud is uneven. In the SVM method, the handcrafted features are also experienced and may not be optimal, thus resulting in a low branch detection rate. Despite the rapid development of deep learning methods ([43–47]), they have high requirements for building large-volume, well-labeled, and rich-diverse datasets.

### Extensibility analysis of the proposed SCAG

The transferability of an algorithm is crucial for ensuring its widespread application. The proposed SCAG was therefore evaluated with an open-source point cloud dataset, namely, Pheno4D [48], to show its general applicability. The Pheno4D dataset contains point clouds of 2 different kinds of crops, i.e., maize and tomato. Maize is a monocotyledonous crop that has a relatively simple structure due to its asymmetrically distributed leaves along a single stem. In contrast, tomato is a dicotyledonous crop that has a much more complex structure than soybean, such as multiple branches around the main stem. This work selected 6 different maize and 6 different tomato point clouds to evaluate the transferability performance of the SCAG.

**Fig. 9.**
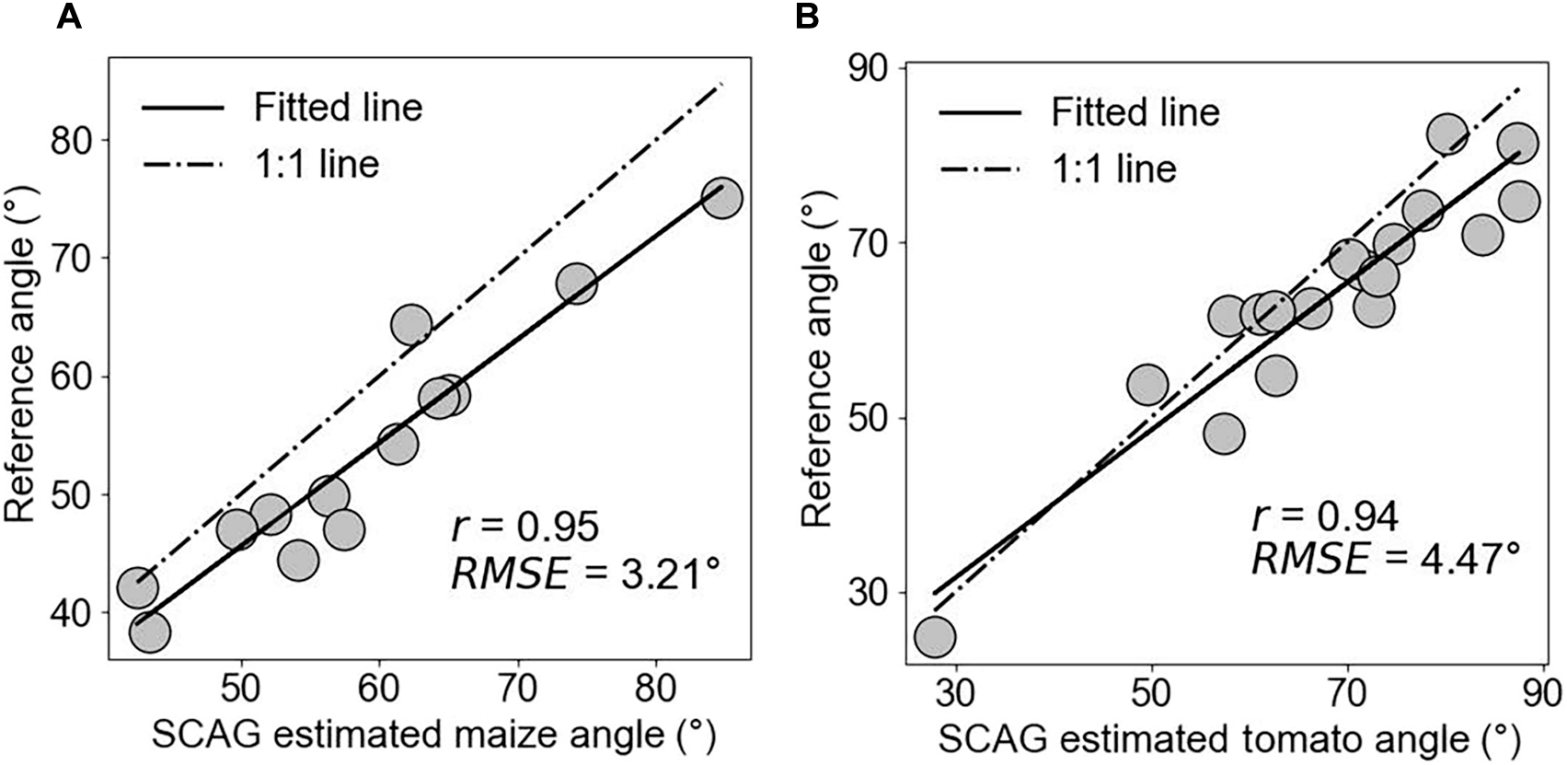
The quantitative results of leaf/branch angle calculations obtained by using the proposed SCAG on 6 (A) maize and (B) tomato point clouds.

As expected, SCAG works well for maize (parameter setting: *H* = 20; *N* = 100; *D* = 30) and tomato (parameter setting: *H* = 5; *N* = 120; *D* = 8) varieties (qualitative results in Fig. S11). The accuracy of the leaf/branch angle calculations was high for maize (*r* = 0.95) and tomato (*r* = 0.94), thus demonstrating its effectiveness across multiple crop types (Fig. 9). The predicted angle values show an overall overestimation in maize, which is mainly caused by the adhesion between the leaf sheath and stem of corn, resulting in the predicted node points being at higher positions than the manually measured node points (Fig. S11).

Therefore, SCAG can be applied not only to soybean for branch angle-related trait extraction but also to other crops, such as maize and tomato, thereby improving architecture-related breeding applications [49,50]. Through a better understanding of how plant architecture affects light penetration, air circulation, and disease susceptibility, breeders can design new varieties that maximize the yield, quality, efficiency [51], and tolerance potential of crops in various environments [52], contributing to global food security and agricultural innovation in the future.

### The application value of the SCAG in ideal soybean type selection

Selecting ideal soybean types is a key step for breeding applications. To construct new traits for ideal soybean type selection, we first extracted 4 basic 3D phenotypes from all 405 varieties in both years, including the average angle (computed by SCAG), canopy width, height, and stem length (Supplemental Materials S5). In this study, we noted that there was an inconsistency of phenotypes between the 2 replications. This inconsistency may be caused by data scanning. One main reason was that the individual plant structure can be damaged when it is transported from the field to the indoors for scanning. The other possible reason is that the scanner has a point resolution of approximately 2 mm, which makes it challenging to characterize thin branches and small stems, especially under occlusions. Therefore, data quality control was conducted based on the basic traits before constructing the new traits. First, if a variety had no sample in any replication of 2 years, it was removed. Second, the consistency of traits between 2 replications in each year was evaluated based on stem length because it had high accuracy (Fig. S7) and was insensitive to plant posture (e.g., tilted or not). If the relative error [53] of the stem length between 2 replications was greater than 30%, the variety was excluded. After data quality filtering, 96 varieties were retained for 2021, and 76 varieties were retained for 2022. Finally, 55 common varieties from both years were identified for further trait exploration.

Second, 11 composite indices were constructed to evaluate density tolerance based on basic traits, including the CHR (a classic density tolerance index), CLR (ratio of canopy width to stem length), LHR (ratio of stem length to height), CAR (ratio of canopy width to average angle), AHR (ratio of average angle to height), ALR (ratio of average angle to stem length), ANR (ratio of average angle to angle number), ACHR (product of average angle and CHR), ACLR (product of average angle and CLR), ANRCHR (product of ANR and CHR) (Supplemental Materials S6). Smaller values for these composite indices meant that the plant looks more compact and should be useful for screening potentially density-tolerant varieties.

The single-year H^2^ [29,54] was calculated for the 96 varieties in 2021 and the 76 varieties in 2022, while the multiyear H^2^ was computed for the 55 varieties across the 2 years. Moreover, 2-way analysis of variance revealed significant differences among varieties for different traits, suggesting the presence of distinct plant types among our materials (Table S2). To better reveal the ability of traits to describe the relative differences among varieties, we evaluated the consistency between the 2 years using the DTW (dynamic time warping) method based on the ranking results of traits for varieties [55]. DTW offers a flexible alternative that does not require data to adhere to specific distribution assumptions and enables the repeatability assessment of trends despite overall differences in absolute (mean) values. A smaller DTW indicates better consistency between 2 sequences. All indices were normalized to the range of 0 to 1 using the min–max scaling method before calculating DTW. The DTW of 2 series of data may be influenced by their series order (the variety number in this study). However, the relative relationship between DTW and different traits was not influenced by the variety order (Table S3), making it suitable for comparing the repeatability of different traits across years. Overall, we expected to screen traits with higher H^2^ and smaller DTW, which should be more robust in evaluating the attributes of density tolerance across years and may be more valuable in future genetic applications.

**Fig. 10.**
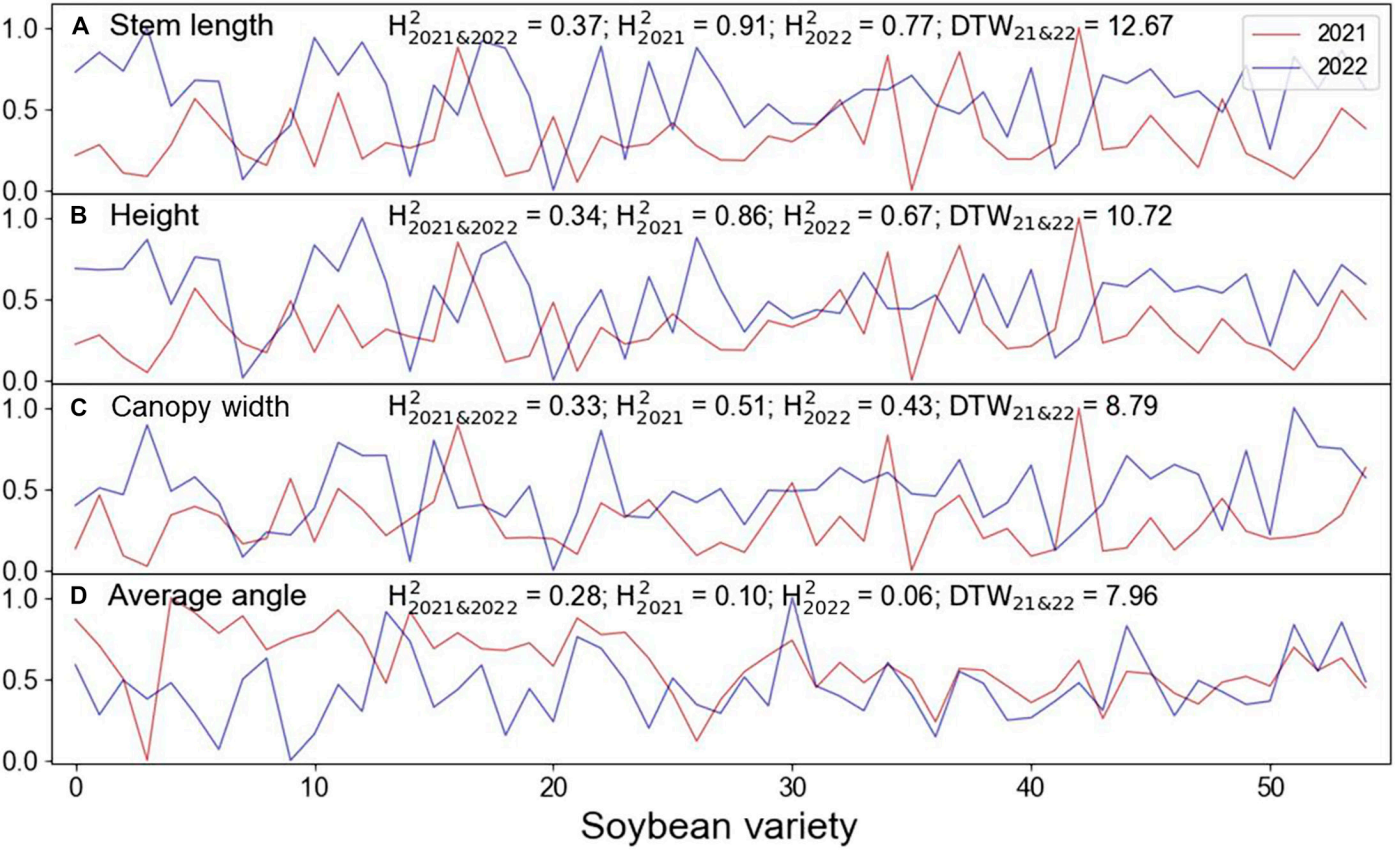
The repeatability (DTW) of (A) stem length, (B) height, (C) canopy width, and (D) average angle across 2 years was compared for 55 soybean types, and their heritability (H^2^) was assessed. Among them, $H_{2021\&2022}^{2}$ exhibited broad-sense heritability between the 2 years. $H_{2021}^{2}$ showed broad-sense heritability in 2021. $H_{2022}^{2}$ showed broad-sense heritability in 2022. The vertical axis of each subplot represents the normalized value of each index, and the horizontal axis represents the different soybean varieties.

Heritability (H^2^) and repeatability (DTW) analyses revealed that stem length and height had better H^2^ values than canopy width and average angle (Fig. 10). Notably, the relatively high H^2^ for stem length may be a result of data quality control. Moreover, different traits had similar repeatability between the 2 years, although the average angle had the best (smallest) DTW.

The analysis of the 11 composite indices showed that the AHR and ALR were clearly better than the CHR in H^2^, while the ANR and CAR were similar to the CHR. All of these angle-related indices had similar repeatability to the CHR (i.e., similar DTW). In contrast, we found that the stem length-related traits LHR and CLR had lower heritability and worse DTW than the CHR for the selection of density-tolerant varieties (Fig. S8). Furthermore, composite indices can be further combined to construct other new traits that are marginally better than the CHR, such as the ACHR and ACLR (Fig. S9), but simple traits are usually preferred among useful traits in practice.

**Fig. 11.**
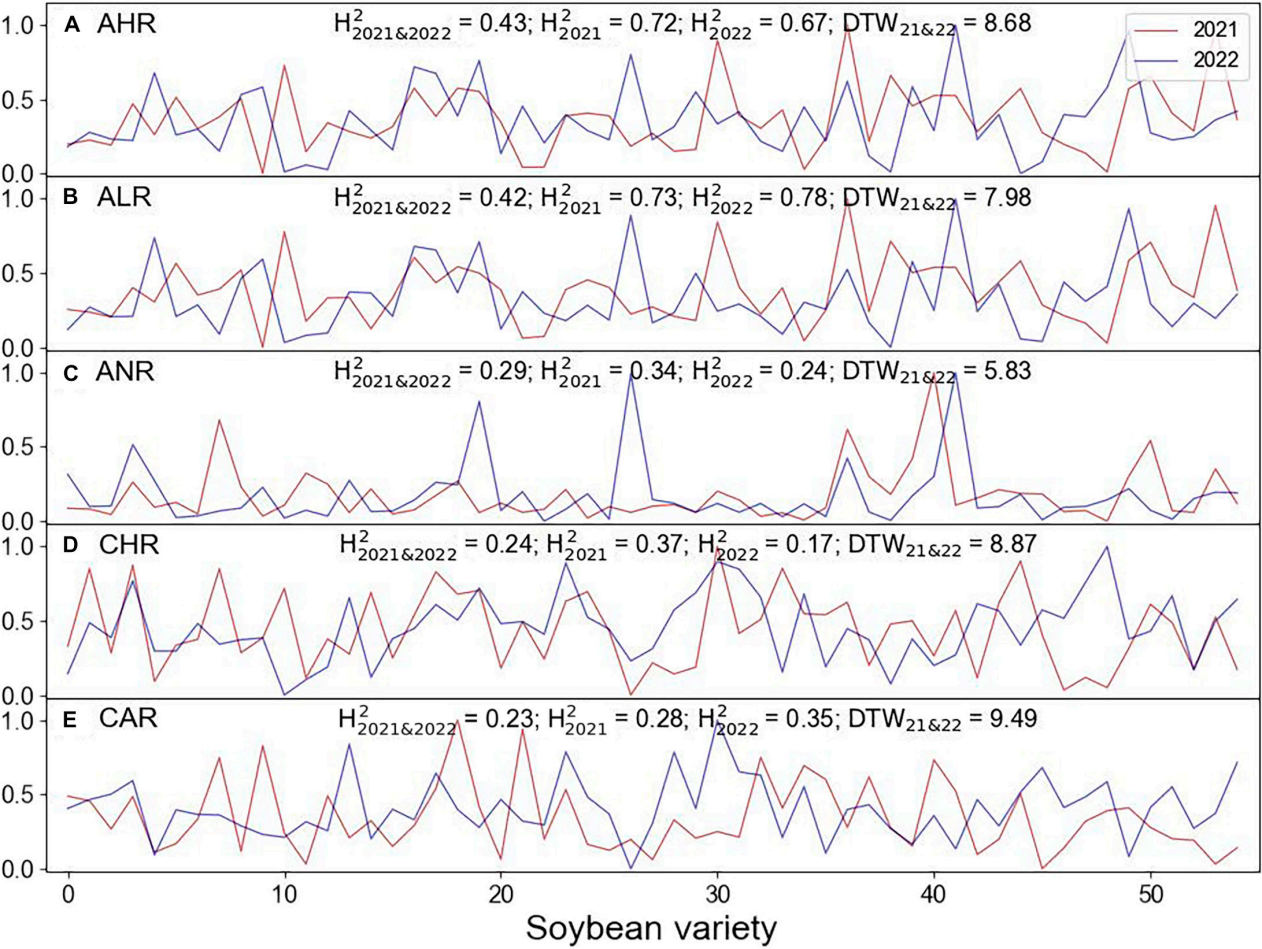
Comparison of the repeatability (DTW) of (A) AHR, (B) ALR, (C) ANR, (D) CHR, and (E) CAR for 55 soybean varieties in 2021 (red line) and 2022 (blue line) and assessment of their heritability (H^2^). $H_{2021\&2022}^{2}$ showed broad-sense heritability between the 2 years. $H_{2021}^{2}$ showed broad-sense heritability for 2021. $H_{2022}^{2}$ showed broad-sense heritability for 2022.

The application value of the SCAG holds great potential for enhancing crop development and agricultural productivity. One important application of SCAG is its ability to screen for potentially ideal plant architecture. Different crops have distinct branch angle preferences, with some requiring more upright architecture to optimize light interception and promote yield production [56]. This study explored novel angle-related traits (e.g., AHR and ALR) that have better heritability (higher H^2^) and repeatability (lower DTW) across years for evaluating density-tolerant varieties (Fig. 11). These novel traits hold potential value for selecting ideal plant types and screening germplasm resources [4,57–61].

### Contributions and implications

This work proposed a biologically inspired method for soybean branch detection and angle calculation based on point clouds. The proposed SCAG incorporates the idea of logical judgment based on continuous changes in the number of branches and stratification, clustering, and growth technologies. The proposed SCAG achieves high branch angle calculation accuracy (*r* = 0.84) and outperforms 2 other branch angle extraction algorithms. Moreover, the proposed SCAG is insensitive to hyperparameters and is suitable for other crops, such as maize and tomato. Moreover, the SCAG-predicted angles are valuable for selecting heritable and repeatable traits to identify density-tolerant varieties. Additionally, diverse datasets (Soybean3D) and source code, such as for deep learning-based branch detection, are publicly available for future studies.

Despite the aforementioned contributions, this work also reveals some implications to be considered in future studies. These implications primarily revolve around data collection and sample quality. First, point cloud data collection is time-consuming due to transportation and scanning. One way to improve the efficiency of 3D data acquisition is to use 3D reconstruction techniques from high-resolution images based on new techniques, such as neural radiance fields [62], which is much less expensive but still challenging in the field. Second, the point cloud accuracy needs to accurately describe the branch thickness. It is worth further exploring how point cloud quality affects the performance of a geometry-based method. In addition, missing data problems are common due to factors such as leaf occlusion, which can be alleviated by introducing data reconstruction algorithms, such as 3D model-based methods [63,64] and 3D geometry-based methods [65,66], during the data preprocessing steps. The underdetection of flattened/broken branches may require a combination of upward and downward detection results or the use of state-of-the-art deep learning methods [67] that may have a better ability to capture long-range/global relationships of branches. The deep learning methods may also be beneficial for detecting small branch targets due to their multiscale feature embedding and attention mechanisms ([45,46,68]). Third, the detected branches may contain petioles that are directly connected to the stem. Discriminating petioles and branches that are both connected to stems is challenging based on the precision of the current data. Once a plant point cloud containing leaves, petioles, branches, and stems can be collected completely, we could use graph theory and technology to discriminate among different organs [69]. Finally, although high-throughput phenotyping has been widely used in genetic analysis, we should be careful about the impact of phenotypic measurement errors on genetic analysis, which are not only random effects but also may be partially under genetic control [70,71].

## Conclusion

Branch detection and angle calculations are important prerequisites for quantifying plant structure and selecting ideal plant types that are important for achieving the soybean Green Revolution. In this work, a novel algorithm (SCAG) for branch detection and angle calculation is proposed. The proposed SCAG achieves good results in terms of the *recall* (0.81), *precision* (0.73), and *F-score* (0.77) for branch detection, as well as *r* (0.84) for angle calculation. The proposed SCAG outperforms the SVM (*F-score* = 0.53) and DB (*F-score* = 0.55) methods, while their angle calculation accuracies are similar. Additionally, the proposed SCAG has proven to be insensitive to parameter *N* within the range of 20 to 140 cm, as well as parameter *D* within the range of 1.5 to 4.0 cm, which are used for node point detection and angle calculation, respectively. Although the proposed SCAG is affected by parameter *H*, the influence pattern is clear, and the optimal values are usually located around the branch diameter. Additionally, the generalization ability of the proposed SCAG for other crops was verified by selecting representative maize (*r* = 0.95) and tomato (*r* = 0.94) point clouds. Finally, we proposed new indices (i.e., AHR and ALR) for screening density-tolerant soybean varieties using SCAG-predicted angles. While we emphasized the contributions of the proposed novel algorithm, we also hope to promote future studies that focus on data quality restoration and the development of artificial general intelligence algorithms by sharing our research dataset, algorithms, and software.

## Data Availability

The Soybean3D datasets, source code, software, and other supporting data are openly available on GitHub (https://github.com/Jinlab-9AiPhenomics/SCAG_PlantAngleExtractor).
