# Supplementary material for: SCAG: A Stratified, Clustered, and Growing-Based Algorithm for Soybean Branch Angle Extraction and Ideal Plant Architecture Evaluation
Source: Plant Phenomics. 2024 Jul 23;6:0190. doi: 10.34133/plantphenomics.0190 (PMC11265809; doi:10.34133/plantphenomics.0190)
Supplement: Supplementary 1 — Tables S1 to S3 Supplemental Materials S1 to S10 [file plantphenomics.0190.f1.docx]

SCAG: A stratified, clustered, and growing-based algorithm for soybean branch angle extraction and ideal plant architecture evaluation

Songyin Zhang^1^, Yinmeng Song^1,2^, Ran Ou^1,2^, Yiqiang Liu^1,3^, Shaochen Li^1^, Xinlan Lu^1^,

Shan Xu^1^, Yanjun Su^4^, Jiang Dong^1,2,3^, Yanfeng Ding^1,2,3^, Haifeng Xia^5^, Qinghua Guo^6^, Jin Wu^7^, Jiaoping Zhang^1,2^,

Shichao Jin ^1, 3^ *

^1^ Crop Phenomics Research Centre, Academy for Advanced Interdisciplinary Studies, Collaborative Innovation Centre for Modern Crop Production co-sponsored by Province and Ministry, State Key Laboratory of Crop Genetics and Germplasm Enhancement, Nanjing Agricultural University, Nanjing 210095, China

^2^ National Center for Soybean Improvement, Key Laboratory for Biology and Genetic Improvement of Soybean (General, Ministry of Agriculture), College of Agriculture, Nanjing Agricultural University, Nanjing 210095, China

^3^ Sanya Research Institute of Nanjing Agriculture University, Sanya 572024, China

^4^ State Key Laboratory of Vegetation and Environmental Change, Institute of Botany, Chinese Academy of Sciences, Beijing 100093, China

^5^ School of Automation, Southeast University, Nanjing 210096, China

^6^ Institute of Remote Sensing and Geographic Information System, School of Earth and Space Sciences, Peking University, Beijing 100871, China

^7^ Division for Ecology and Biodiversity, School of Biological Sciences, The University of Hong Kong, Pokfulam Road, Hong Kong, China

*** Corresponding authors**

[jinshichao1993@gmail.com](mailto:jinshichao1993@gmail.com); jschaon@njau.edu.cn

The following Supporting Information is available for this article:

[SCAG: A stratified, clustered, and growing-based algorithm for soybean branch angle extraction and ideal plant architecture evaluation 1](#_Toc163925464)

[**Supplemental material S1** 3](#_Toc163925465)

[**Diversity analysis of the *Soybean3D* dataset** 3](#_Toc163925466)

[**Supplemental material S2** 7](#_Toc163925467)

[**Branch angle extraction of different methods** 7](#_Toc163925468)

[**Supplemental material S3** 8](#_Toc163925469)

[**Density-based method for branch angle calculation** 8](#_Toc163925470)

[**Supplemental material S4** 10](#_Toc163925471)

[**Support vector machine-based method for branch angle calculation** 10](#_Toc163925472)

[**Supplemental material S5** 12](#_Toc163925473)

[**The calculation algorithm for three basic traits.** 12](#_Toc163925474)

[**Supplemental material S6** 15](#_Toc163925475)

[**Selection of density-tolerance indices** 15](#_Toc163925476)

[**Supplemental material S7** 18](#_Toc163925477)

[**Comparison of branch angle calculation methods** 18](#_Toc163925478)

[**Supplemental material S8** 19](#_Toc163925479)

[**The qualitative results of SCAG in other crops** 19](#_Toc163925480)

[**Supplemental material S9** 21](#_Toc163925481)

[The two-way analysis of variance (ANOVA) of different traits 21](#_Toc163925482)

[**Supplemental material S10** 22](#_Toc163925483)

[**Robustness of the DTW method for ranking varieties** 22](#_Toc163925484)

**Supplemental material S1**

**Diversity analysis of the *Soybean3D* dataset**

The LiDAR data from multiple stations of TLS was registered using the *Faro Scene* software. The point cloud of the plant area was segmented and denoised by using the *CloudCompare* software. In this work, 152 varieties of soybean samples in 2021 (hereafter referred to as the *Soybean3D* dataset) were selected for algorithm development. To verify the accuracy of the proposed algorithm, the node points of the *Soybean3D* dataset were labeled manually, and the branch angles were carefully measured. Specifically, the points of each plant point cloud were given a new attribute field, i.e., class. The node points were labeled as class 1, and the other points were labeled as class 0 in *CloudCompare* software. The soybean branch angles were manually measured in the point cloud based on the node and branch points that were visually selected based on the field survey experiences. Notably, the locations of branch points were around 2~3 cm above the determined node points in this study.

The 152 individuals in *Soybean3D* dataset provided enough diversity in terms of plant architectures and data quality by considering six indicators, including average nearest point distance (NPD), plant height, canopy width, the ratio of canopy width to plant height (CHR), angle number, and average branch angle (Fig. S1). The NPD values ranged between 0.36 mm and 1.12 mm, with a mean value of 0.75 mm and a standard deviation value of 0.12 (Fig. S1a). The plant height was the vertical distance between the lowest and the highest point. The canopy width was defined as the diagonal distance of the bounding box of points projected from top to down. The plant height and canopy width ranged between 22.43cm-108.48cm and 5.67cm-47.91cm, respectively. The CHR represented the plant structure. The lower the value of CHR, the plant structure tended to be narrower and taller. The CHR ranged between 0.1 to 0.6. The angle number denoted the angles contained in each plant. It also showed the diversity of the samples and ranges between 1 and 11, with a mean of 3.78 and a standard deviation of 1.88. The average branch angle represented the average angle in each plant. The average branch angle ranged between 26.42° and 93.72°, with a mean value of 48.83° and a standard deviation of 12.31°. These angle attributes showed that the plant structures are diverse and representative.


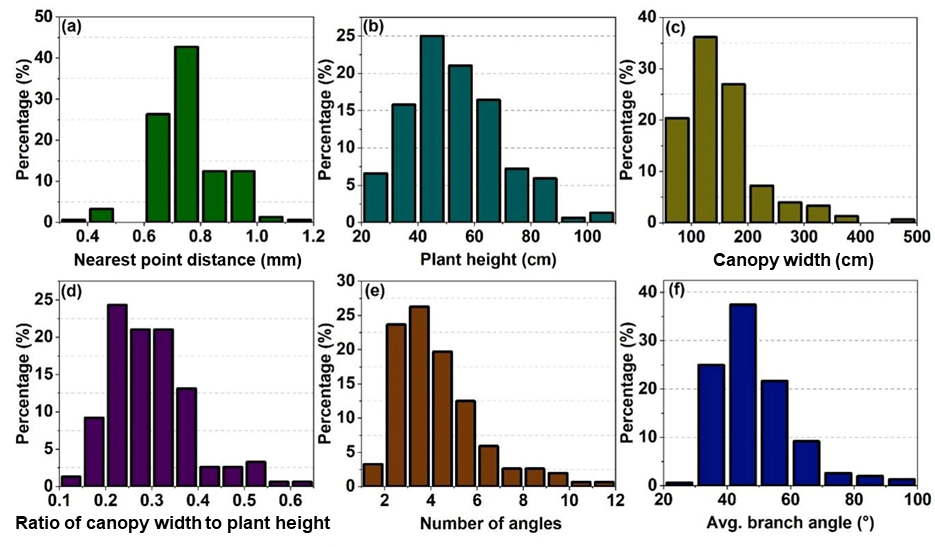


**Fig. S1.** The frequency distributions of the samples in *Soybean3D* dataset. (a) Nearest point distance (NPD). (b) Plant height. (c) Canopy width. (d) The ratio of canopy width to plant height (CHR). (e) The number of angles. (f) Average branch angle. Each attribute (y-axis of each subfigure) was calculated for each individual plant sample, and the frequency distribution was derived for all samples in *Soybean3D* dataset.

To evaluate the robustness of the proposed algorithm, the *Soybean3D* dataset was grouped into different difficulty levels based on the mean values of angle number, branch angle, and nearest point distance (NPD). When the angle number was large and the branch angle was small, the plant architecture was complex. Meanwhile, when the NPD was large, the data quality was bad and made the dataset more complex ([Table S1](file:///D:\software\wechat-files\WeChat%20Files\wxid_6t9knqp8jif021\FileStorage\File\2023-05\Zhang_Manuscript-0406.docx)). The simple, medium, and complex groups contained 50, 51, and 51 soybean varieties, respectively. The simple group had the least number of angles and the largest average branch angle. The average angle number of simple, medium, and complex groups were 3.16, 3.59, and 4.53, respectively. The mean average branch angle of these groups was 51.63°, 48.90°, and 45.80°, respectively. In other words, the simple group had fewer angle numbers and bigger branch angles, which induced fewer occlusions during the data acquisition process, thus ensuring highly accurate branch detection and angle calculation.

**Table S1.** The statistical properties of three groups of samples with different complexities were used in this experiment. CHR denoted the ratio of canopy width to plant height, and NPD represented the average neighbor point distance.

| **Groups** | **Sample number** | **Number of branch angles** | | | **Avg. branch angle (°)** | | | **CHR** | | | **NPD (mm)** | | |
| --- | --- | --- | --- | --- | --- | --- | --- | --- | --- | --- | --- | --- | --- |
|  |  | Min | Max | Mean | Min | Max | Mean | Min | Max | Mean | Min | Max | Mean |
| **Simple** | 50 | 1 | 7 | 3.16 | 31.24 | 93.72 | 51.63 | 0.15 | 0.51 | 0.28 | 0.36 | 0.91 | 0.72 |
| **Medium** | 51 | 1 | 7 | 3.59 | 30.56 | 88.63 | 48.90 | 0.10 | 0.60 | 0.28 | 0.43 | 1.02 | 0.77 |
| **Complex** | 51 | 1 | 11 | 4.53 | 26.42 | 83.46 | 45.80 | 0.15 | 0.56 | 0.32 | 0.44 | 1.12 | 0.76 |

**Supplemental material S2**

**Branch angle extraction of different methods**

**
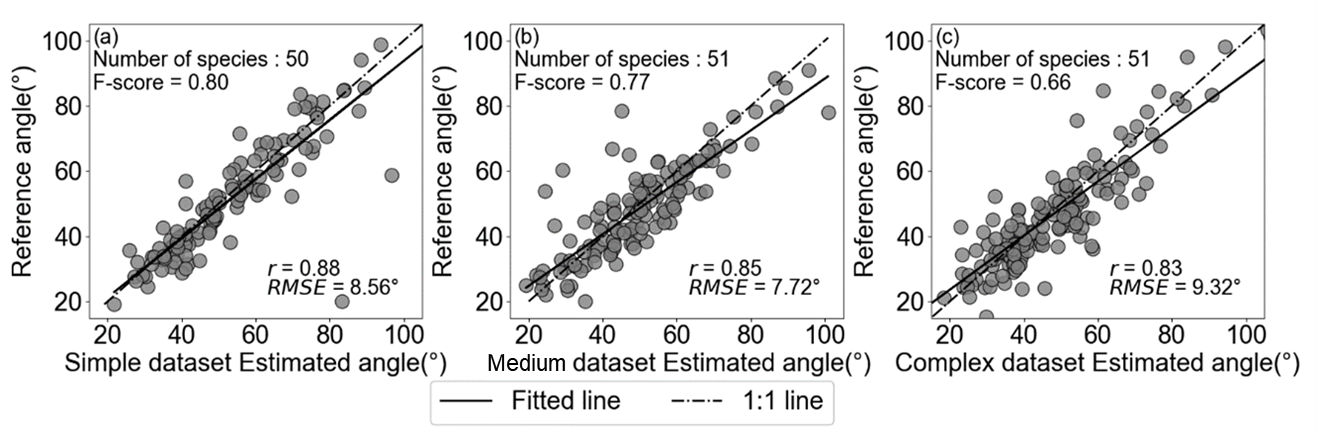
**

**Fig. S2.** A comparison of the branch angle extraction based on the proposed SCAG and manual measurement using (a) simple, (b) medium, and (c) complex groups in the *Soybean3D* dataset. There are 574 branch angles (i.e., scattered points) in the whole dataset. There was an obviously overestimated sample in subfigure (a). We have doubly checked the raw data and found that the overestimation was mainly caused by the missing points at the branch node location in the sample.

**Supplemental material S3**

**Density-based method for branch angle calculation**

The density-based method was proposed by assuming that the point densities around the node points were higher than the other locations. To locate these node points, a density-based method with two parameters, radius (*Rad*) and proportion (*Pro*), was proposed. *Rad* was used for calculating the point density by counting the number of points within the radius of each point. *Pro* was used for sorting the top *Pro* percent points based on the point density from high to low. In branch detection, both *Rad* and *Pro* affect *Recall*, *Precision*, and *F-score* (Fig. S3). If *Rad* was too small, the radius region only had a few points, thus making it hard to differentiate the noise and signal points. If *Rad* was too large, the radius region may include many branch points, thus making it hard to differentiate between the node and other points. The *Rad* parameter was suggested to be set in such a way that it can exactly include the node of a branch. The best *Rad* was around 1cm, and the best *Pro* was around 0.2, as revealed by the best *F-score* parameter analysis.

**
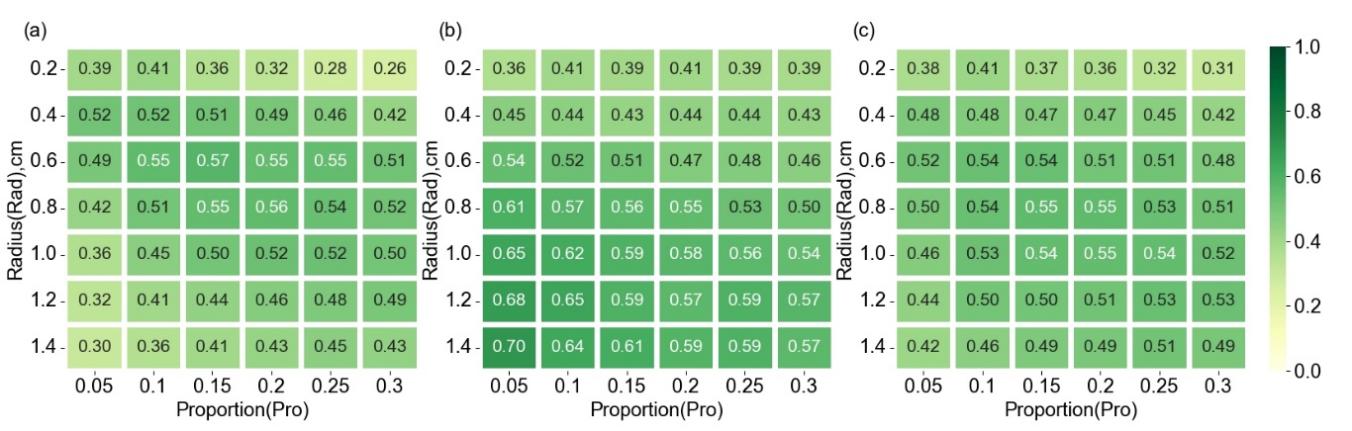
**

**Fig.** **S3.** The parameter sensitivity analysis of branch detection for the density-based method. (a), (b), and (c) represented *Recall*, *Precision*, and *F-score* of branch detection under various parameter combinations of radius (*Rad*) and proportion (*Pro*).

In angle calculation, the parameter that affected the result of angle calculation was slice depth (*D*), which was the same as the proposed SCAG. The value of *r* remained stable across different parameter combinations. The *r* was relatively higher when *D* was around 2.0~3.0cm (Fig. S4), which was close to the height difference between the manually measured branch points and node points.

**
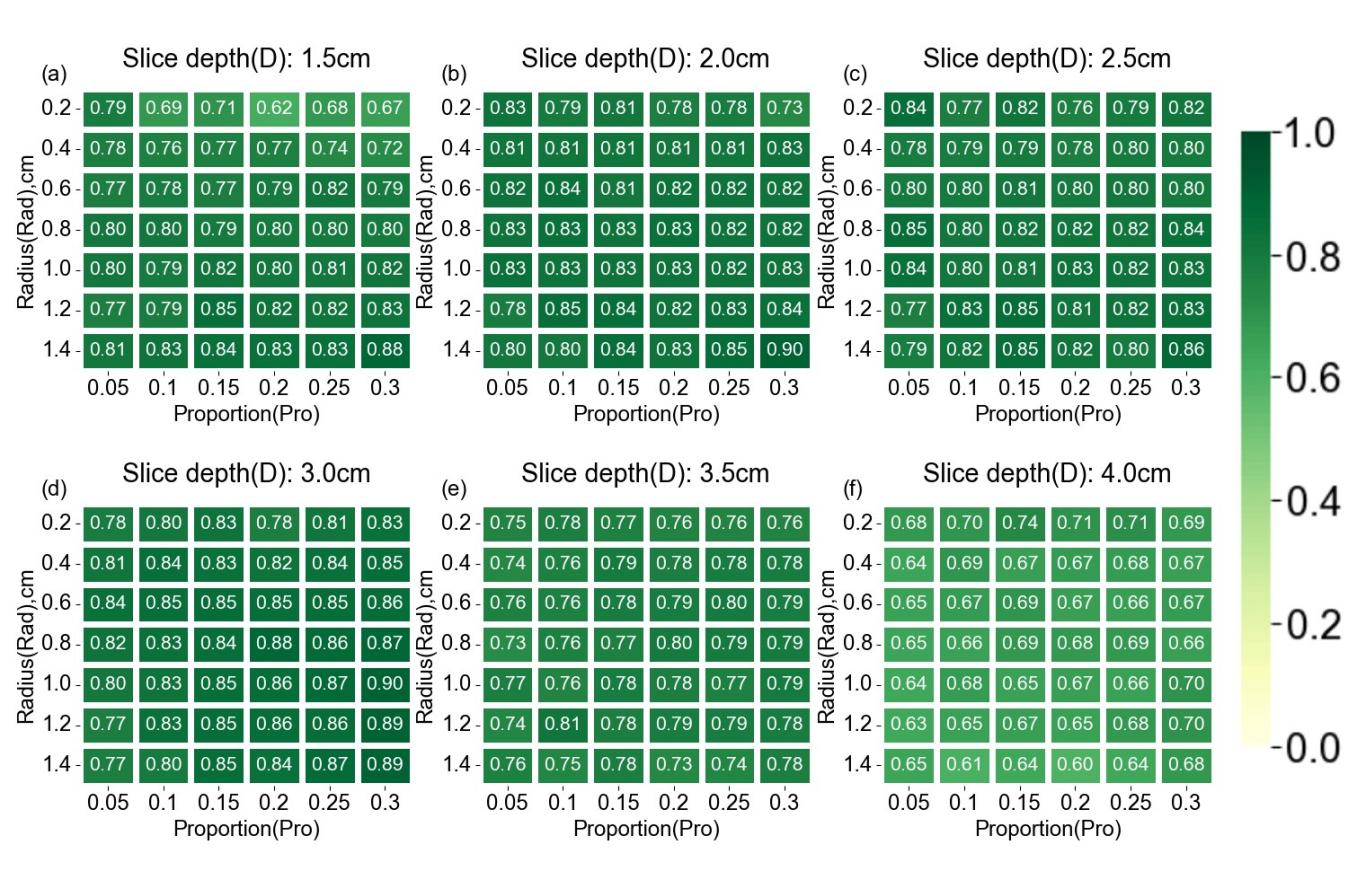
**

**Fig. S4.** The parameter sensitivity analysis of branch angle calculation for the density-based method. The title of each subplot denoted the value of parameter *D*, and the horizontal and vertical coordinates of each subplot represent parameters *Pro* and *Rad*, respectively.

**Supplemental material S4**

**Support vector machine-based method for branch angle calculation**

Support vector machine (SVM) was a widely used method for classification based on small samples. The model took manually designed features as input, which included raw coordinates (i.e., X, Y, Z), feature vectors (i.e., $e_{1}, e_{2}, e_{3}$), linear features ($L_{\lambda}$), planar features ($P_{\lambda}$), sphericity ($S_{\lambda}$), curvature variation ($C_{\lambda}$), trace ($T_{\lambda}$), and anisotropy ($A_{\lambda}$). Because multi-scale features have been proved useful in point classification, we calculated features of each point within its neighborhood points in different ranges (i.e., 1cm, 5cm, 10cm, 15cm, and 20cm). The feature number (*Fea*) was used to represent the number of features for each point. In this study, the *Fea* for different ranges were 12 ($3+1\times9$), 21 ($3+2\times9$), …, and 48 ($3+5\times9$), which were compared to see the influence of *Fea* on SVM based angle detection. The points in the node region were labeled as class 1, and the other points were labeled as class 0 to generate a training set using 50 individual samples. The SVM was trained with an RBF kernel. The results showed that increasing *Fea* does not affect the *Precision*, *Recall*, and *F-score* of the branch detection significantly. The best *F-score* was around 0.53 (Fig. S5).

|  | $e_{i}=\frac{\lambda_{i}}{\sum\lambda_{i}} , i\in\left[ 1,2,3 \right]$ | (S1) |
| --- | --- | --- |
|  | $L_{\lambda}= \frac{\lambda_{1}-\lambda_{2}}{\lambda_{1}}$ | (S2) |
|  | $P_{\lambda}=\frac{\lambda_{2}-\lambda_{3}}{\lambda_{1}}$ | (S3) |
|  | $S_{\lambda}= \frac{\lambda_{3}}{\lambda_{1}}$ | (S4) |
|  | $C_{\lambda}=\frac{3\lambda_{3}}{\lambda_{1}+\lambda_{2}+\lambda_{3}}$ | (S5) |
|  | $T_{\lambda}=\frac{2}{\pi}arctan(\lambda_{1}+\lambda_{2}+\lambda_{3})$ | (S6) |
|  | $A_{\lambda}= \frac{\lambda_{1}-\lambda_{3}}{\lambda_{1}}$ | (S7) |

where, $\lambda_{i}$ represented the eigenvalues calculated by principal component analysis.

The parameter that affected the results of angle calculation was slice depth (*D*), which was the same as the proposed SCAG and DB. The *r* was also relatively higher when *D* was around 2.5 cm (Fig. S5), which was close to the height difference between the manually measured branch points and node points.


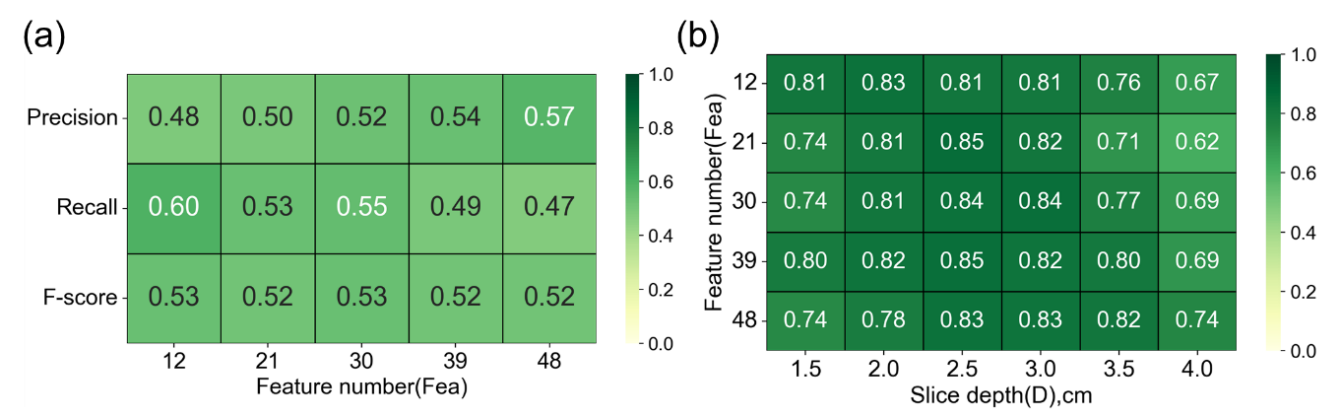


**Fig. S5.** The parameter sensitivity analysis of the SVM-based method during (a) branch detection and (b) branch angle calculation.

**Supplemental material S5**

**The calculation algorithm for three basic traits.**

In addition to calculating the branch angle, this study also computed three other basic traits: stem length, height, and canopy width (Fig. S6). Stem length was the accumulated length calculated by connecting the node points from bottom to up. First, all node points are connected into segmented lines in ascending order. Then, stem points around each segmented line were identified and extracted. Finally, these extracted points were used to calculate the median point for each segment. These median points were connected into more smoothed lines, and these segmented lines were used to determine the total length.

Height and canopy width were obtained by calculating the minimum bounding box of the plant. The bounding box’s height was used as plant height. The bounding box's length and width were used to calculate the diagonal length and used as the canopy width. This study randomly selected 30 samples for algorithm accuracy verification, resulting in correlation coefficients of 0.97, 0.99, and 0.95 for stem length, height, and canopy width respectively (Fig. S7). This demonstrated the algorithm's high precision.

**
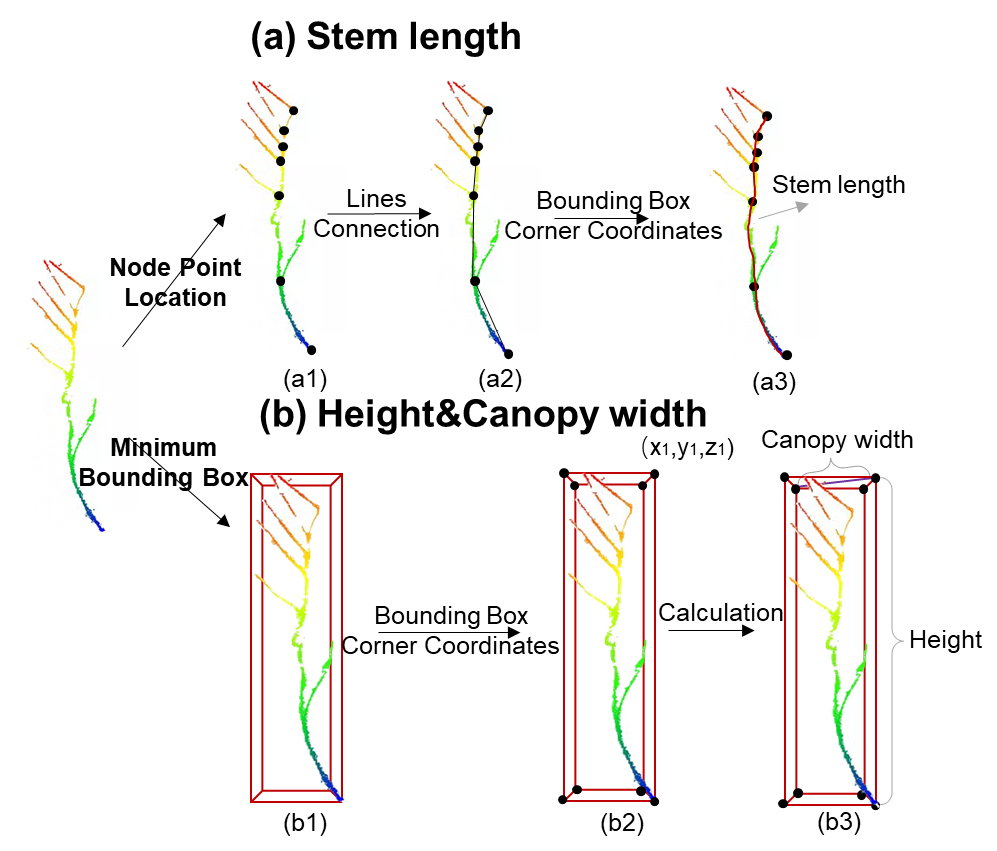
**

**Fig. S6.** Stem length, height, and canopy width calculation process diagram. (a) Stem length calculation diagram: (a1) The black dots represented the node points found using the node point location. (a2) Connect the node points with straight lines. (a3) Extract the adjacent points of these lines to fit and calculate the stem length. (b) Height and canopy width calculation process diagram: (b1) Calculate the minimum bounding box of the plant. (b2) Calculate the corner points of the bounding box. (b3) The bounding box’s height was used as the plant height, and the diagonal length of the bounding box’s bottom rectangle was extracted as the canopy width.

**
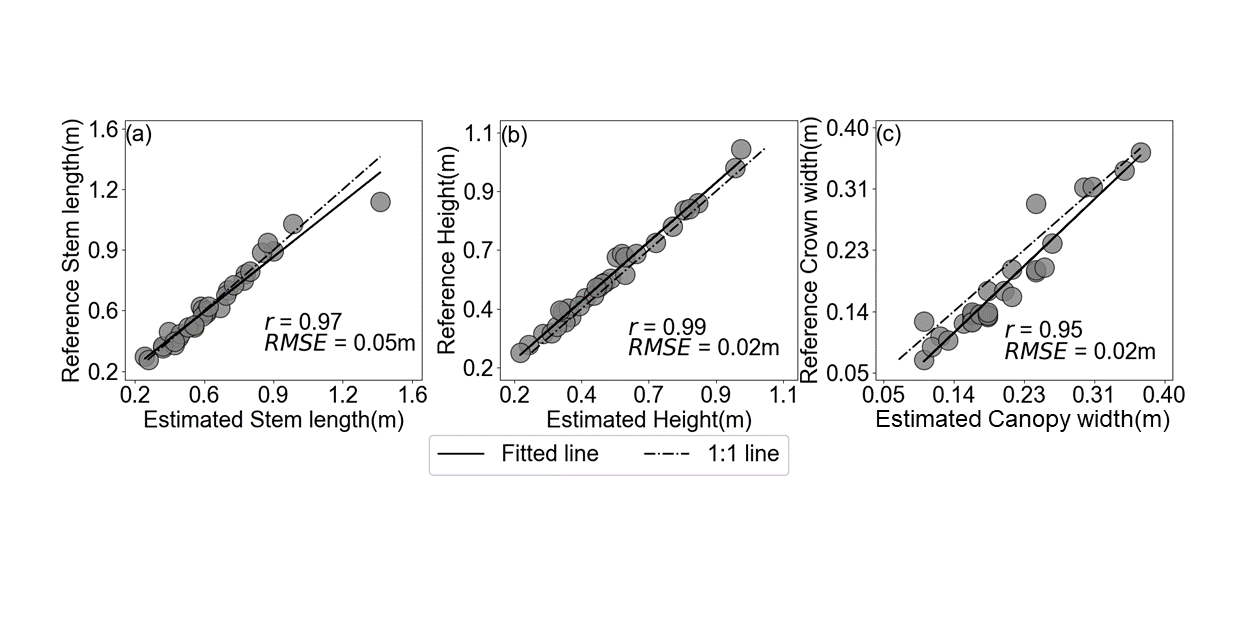
**

**Fig. S7.** Scatter plots of stem length, height, and canopy width algorithmic results.

**Supplemental material S6**

**Selection of density-tolerance indices**

This study constructed 11 indices related to density resistance, including the CHR (ratio of canopy width to height), CLR (ratio of canopy width to stem length), LHR (ratio of stem length to height), CAR (ratio of canopy width to average angle), AHR (ratio of average angle to height), ALR (ratio of average angle to stem length), ANR (ratio of average angle to angle number), ACHR (product of average angle and CHR), ACLR (product of average angle and CLR), ANRCHR (product of ANR and CHR). Among them, CHR was a classical index for characterizing plant architecture. The formulas were as below,

|  | $CHR= \frac{W}{H}$ | (S8) |
| --- | --- | --- |
|  | $CLR= \frac{L}{H}$ | (S9) |
|  | $LHR= \frac{L}{H}$ | (S10) |
|  | $CAR=\frac{W}{cos\alpha_{i}}$ | (S11) |
|  | $AHR= \frac{\sin\alpha_{i}}{H}$ | (S12) |
|  | $ALR= \frac{\sin\alpha_{i}}{L}$ | (S13) |
|  | $ANR= \frac{\alpha_{i}}{N}$ | (S14) |
|  | $ACHR= sin\alpha_{i}*CHR$ | (S15) |
|  | $ACLR= sin\alpha_{i}*CLR$ | (S16) |
|  | $ALHR= sin\alpha_{i}*LHR$ | (S17) |
|  | $ANRCHR= ANR*CHR$ | (S18) |

where$W$ was the canopy width. *H* was the plant height. *L* was the stem length. $\alpha_{i}$ was the average angle. *N* was the angle number.

**
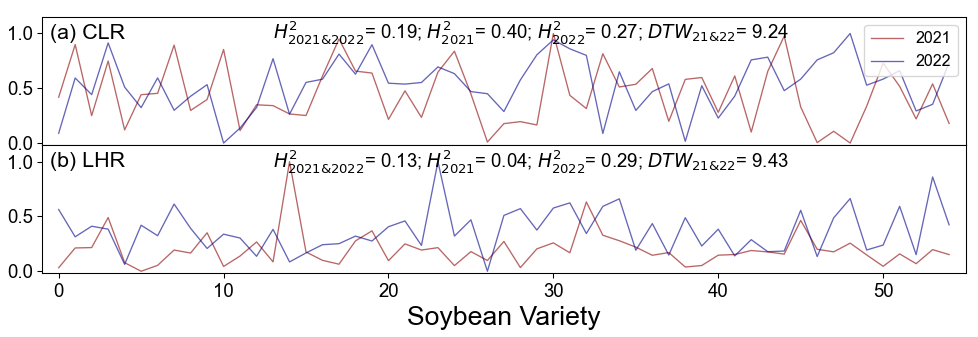
**

**Fig. S8.** The repeatability of the stem length related indices (i.e., LHR and CLR) for 55 soybean varieties in 2021 (red line) and 2022 (blue line), and their heritability (H^2^). Among them, $H_{2021\&2022}^{2}$ was broad-sense heritability between two years. $H_{2021}^{2}$ was broad-sense heritability in 2021. $H_{2022}^{2}$ was broad-sense heritability in 2022. DTW was the repeatability/consistency of the same index between 2021 and 2022. The vertical axis of each subplot was the normalized value of each index, and the horizontal axis was the different soybean varieties.

**
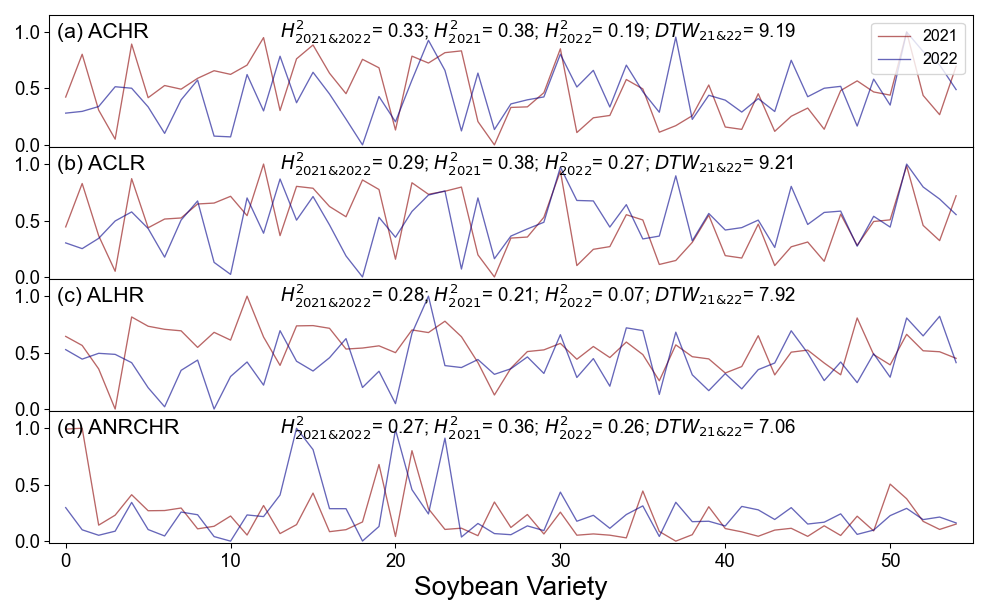
**

**Fig. S9.** The repeatability of indices composited by more than three basic indices for 55 soybean varieties in 2021 (red line) and 2022 (blue line), and their heritability (H^2^). (a) ANRCHR, (b) ACLR, (c) ACHR, (d)ALHR. Among them, $H_{2021\&2022}^{2}$ was broad-sense heritability between two years. $H_{2021}^{2}$ was broad-sense heritability in 2021.$H_{2022}^{2}$ was broad-sense heritability in 2022. DTW was the repeatability/consistency of the same index between 2021 and 2022. The vertical axis of each subplot was the normalized value of each index, and the horizontal axis was the different soybean varieties.

**Supplemental material S7**

**Comparison of branch angle calculation methods**


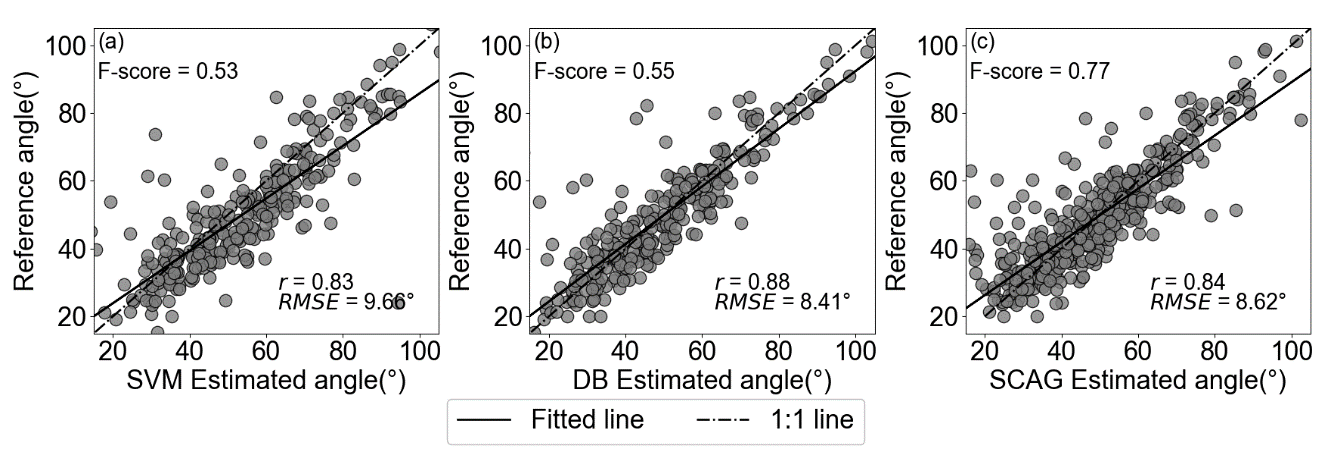


**Fig. S10.** A comparison of branch angle calculation between manual measurement and (a) the SVM method, (b) the DB method, and (c) the proposed SCAG method.

**Supplemental material S8**

**The qualitative results of SCAG in other crops**

The qualitative results showed that the proposed SCAG performed effectively in leaves/branches detection for both maize and tomato. There were certain limitations that have also been reported in the existing studies, such as miss detection of small leaves/branches (the lowest leaves in [Fig. S1](file:///D:\software\wechat-files\WeChat%20Files\wxid_6t9knqp8jif021\FileStorage\File\2023-05\Zhang_Manuscript-0406.docx)1 a1-a3, a5-a6) and the bend or flattened leaves/branches ([Fig. S11](file:///D:\software\wechat-files\WeChat%20Files\wxid_6t9knqp8jif021\FileStorage\File\2023-05\Zhang_Manuscript-0406.docx) a4, b1, b6).

**
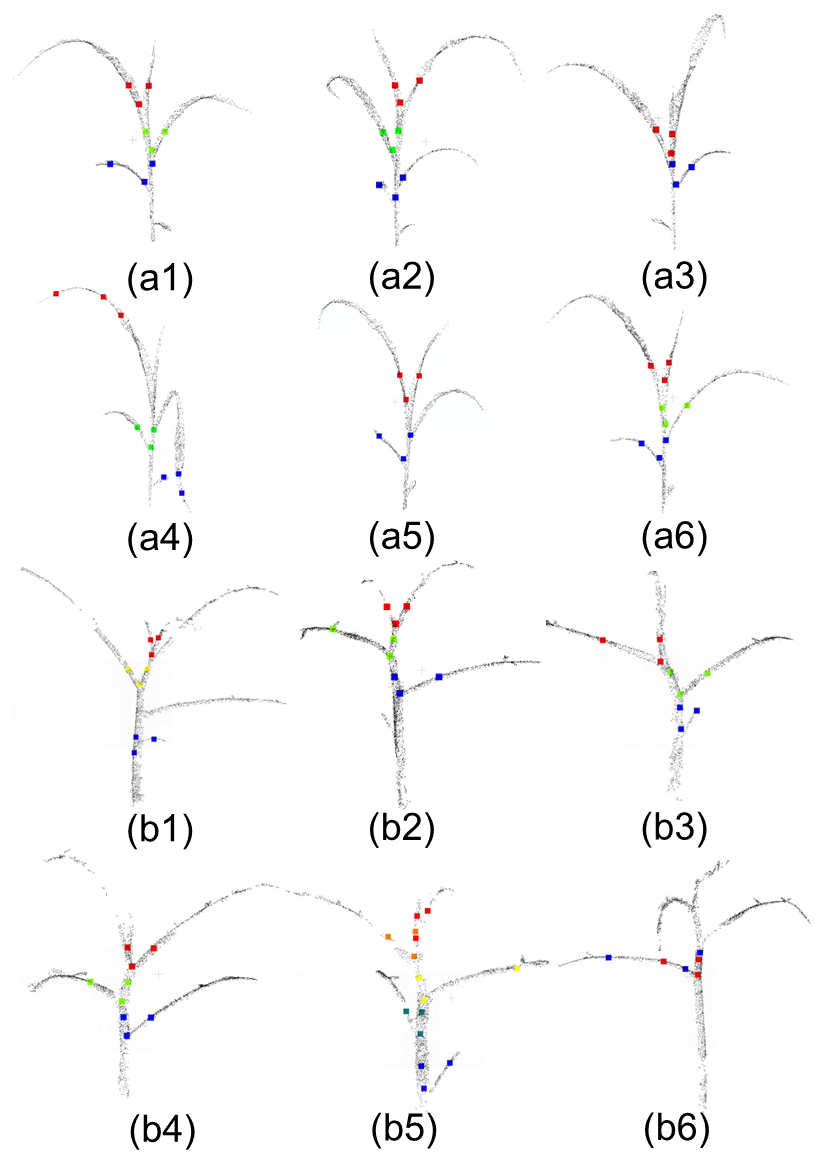
**

**Fig. S11.** The qualitative results of branch detection were obtained by using the proposed SCAG algorithm on (a1) - (a6) maize and (b1) - (b6) tomato crops with regard to representative plant architecture. The three points in different colors on each soybean crop represented one node point and two branch points of a branch detected by the proposed SCAG method.

**Supplemental material S9**

The two-way analysis of variance (ANOVA) of different traits

The ANOVA results indicated that: (1) Significant differences exist among varieties for different traits, suggesting the presence of distinct plant types among our materials; (2) Among different years, these traits exhibit significant differences, indicating that plant type traits are influenced by varying environmental conditions across years; (3) The interaction results between varieties and years are also significant

**Table S2.** The analysis of variance (ANOVA) results for different traits. *Val* represents varieties. *Rep* represents replications. The *df* represents degree of freedom. The *sum_sq* represents sum of squares. The *mean_sq* represents mean squares. *PR(>F)* represents probability of F-statistic. "-" indicates that Residual is not involved in the F-test.

| **Stem length** | df | sum_sq | mean_sq | F | PR(>F) |
| --- | --- | --- | --- | --- | --- |
| Var | 54 | 2.008153 | 0.037188 | 5.695299 | 7.77E-15 |
| Year | 1 | 2.229361 | 2.229361 | 341.42383 | 2.28E-35 |
| Var:Year | 54 | 2.878017 | 0.053297 | 8.162308 | 1.69E-20 |
| Year:Rep | 1 | 0.000133 | 0.000133 | 0.02035 | 8.87E-01 |
| Residual | 109 | 0.711726 | 0.00653 | - | - |
| **Canopy width** | df | sum_sq | mean_sq | F | PR(>F) |
| Var | 54 | 0.23059 | 0.00427 | 1.933057 | 1.86E-03 |
| Year | 1 | 0.261154 | 0.261154 | 118.22099 | 4.30E-19 |
| Var:Year | 54 | 0.230878 | 0.004276 | 1.935469 | 1.83E-03 |
| Year:Rep | 1 | 0.000555 | 0.000555 | 0.251218 | 6.17E-01 |
| Residual | 109 | 0.240785 | 0.002209 | - | - |
| **Height** | df | sum_sq | mean_sq | F | PR(>F) |
| Var | 54 | 1.599131 | 0.029614 | 3.767551 | 2.10E-09 |
| Year | 1 | 1.099158 | 1.099158 | 139.83919 | 2.93E-21 |
| Var:Year | 54 | 2.30013 | 0.042595 | 5.419103 | 4.06E-14 |
| Year:Rep | 1 | 0.008193 | 0.008193 | 1.042311 | 3.10E-01 |
| Residual | 109 | 0.856757 | 0.00786 | - | - |
| **Average angle** | df | sum_sq | mean_sq | F | PR(>F) |
| Var | 54 | 5481.6458 | 101.51196 | 1.412701 | 6.48E-02 |
| Year | 1 | 2564.8193 | 2564.8193 | 35.693562 | 2.95E-08 |
| Var:Year | 54 | 4040.6437 | 74.826734 | 1.041334 | 4.21E-01 |
| Year:Rep | 1 | 65.310627 | 65.310627 | 0.908902 | 3.43E-01 |
| Residual | 109 | 7832.3732 | 71.856635 | - | - |

**Supplemental material S10**

**Robustness of the DTW method for ranking varieties**

**Table S3.** The rank of five traits according to DTW under different variety order. SN denoted the species number. SN1 to SN5 represented five types of SN defined randomly.

| Traits | Rank | SN1 | SN2 | SN3 | SN4 | SN5 |
| --- | --- | --- | --- | --- | --- | --- |
| Average angle | 1 | 7.96 | 8.93 | 8.51 | 9.29 | 8.88 |
| Canopy width | 2 | 8.79 | 9.52 | 10.68 | 9.69 | 9.78 |
| Height | 3 | 10.72 | 9.89 | 12.28 | 10.66 | 10.01 |
| Stem length | 4 | 12.67 | 10.86 | 12.95 | 10.69 | 12.31 |
